# Natural language instructions induce compositional generalization in networks of neurons

**DOI:** 10.1038/s41593-024-01607-5

**Published:** 2024-03-18

**Authors:** Reidar Riveland, Alexandre Pouget

**Affiliations:** Department of Basic Neuroscience, University of Geneva, Geneva, Switzerland

**Keywords:** Language, Intelligence, Network models

## Abstract

A fundamental human cognitive feat is to interpret linguistic instructions in order to perform novel tasks without explicit task experience. Yet, the neural computations that might be used to accomplish this remain poorly understood. We use advances in natural language processing to create a neural model of generalization based on linguistic instructions. Models are trained on a set of common psychophysical tasks, and receive instructions embedded by a pretrained language model. Our best models can perform a previously unseen task with an average performance of 83% correct based solely on linguistic instructions (that is, zero-shot learning). We found that language scaffolds sensorimotor representations such that activity for interrelated tasks shares a common geometry with the semantic representations of instructions, allowing language to cue the proper composition of practiced skills in unseen settings. We show how this model generates a linguistic description of a novel task it has identified using only motor feedback, which can subsequently guide a partner model to perform the task. Our models offer several experimentally testable predictions outlining how linguistic information must be represented to facilitate flexible and general cognition in the human brain.

## Abstract (web summary)

Riveland and Pouget model instructed action, showing that shared structure in task and semantic representations allows language to compose practiced skills in novel settings. Models make predictions for neural activity in human language areas.

## Main

In a laboratory setting, animals require numerous trials in order to acquire a new behavioral task. This is in part because the only means of communication with nonlinguistic animals is simple positive and negative reinforcement signals. By contrast, it is common to give written or verbal instructions to humans, which allows them to perform new tasks relatively quickly. Further, once humans have learned a task, they can typically describe the solution with natural language. The dual ability to use an instruction to perform a novel task and, conversely, produce a linguistic description of the demands of a task once it has been learned are two unique cornerstones of human communication. Yet, the computational principles that underlie these abilities remain poorly understood.

One influential systems-level explanation posits that flexible interregional connectivity in the prefrontal cortex allows for the reuse of practiced sensorimotor representations in novel settings^1,2^. More recently, multiple studies have observed that when subjects are required to flexibly recruit different stimulus-response patterns, neural representations are organized according to the abstract structure of the task set^3–5^. Lastly, recent modeling work has shown that a multitasking recurrent neural network (RNN) will share dynamical motifs across tasks with similar demands^6^. This work forms a strong basis for explanations of flexible cognition in humans but leaves open the question of how linguistic information can reconfigure a sensorimotor network so that it performs a novel task well on the first attempt. Overall, it remains unclear what representational structure we should expect from brain areas that are responsible for integrating linguistic information in order to reorganize sensorimotor mappings on the fly.

These questions become all the more pressing given that recent advances in machine learning have led to artificial systems that exhibit human-like language skills^7,8^. Recent works have matched neural data recorded during passive listening and reading tasks to activations in autoregressive language models (that is, GPT^9^), arguing that there is a fundamentally predictive component to language comprehension^10,11^. Additionally, some high-profile machine learning models do show the ability to use natural language as a prompt to perform a linguistic task or render an image, but the outputs of these models are difficult to interpret in terms of a sensorimotor mapping that we might expect to occur in a biological system^12–14^. Alternatively, recent work on multimodal interactive agents may be more interpretable in terms of the actions they take, but utilize a perceptual hierarchy that fuses vision and language at early stages of processing, making them difficult to map onto functionally and anatomically distinct language and vision areas in human brains^15–17^.

We, therefore, seek to leverage the power of language models in a way that results in testable neural predictions detailing how the human brain processes natural language in order to generalize across sensorimotor tasks.

To that end, we train an RNN (sensorimotor-RNN) model on a set of simple psychophysical tasks where models process instructions for each task using a pretrained language model. We find that embedding instructions with models tuned to sentence-level semantics allow sensorimotor-RNNs to perform a novel task at 83% correct, on average. Generalization in our models is supported by a representational geometry that captures task subcomponents and is shared between instruction embeddings and sensorimotor activity, thereby allowing a composition of practice skills in a novel setting. We also find that individual neurons modulate their tuning based on the semantics of instructions. We demonstrate how a network trained to interpret linguistic instructions can invert this understanding and produce a linguistic description of a previously unseen task based on the information in motor feedback signals. We end by discussing how these results can guide research on the neural basis of language-based generalization in the human brain.

## Results

### Instructed models and task set

**Fig. 1:**
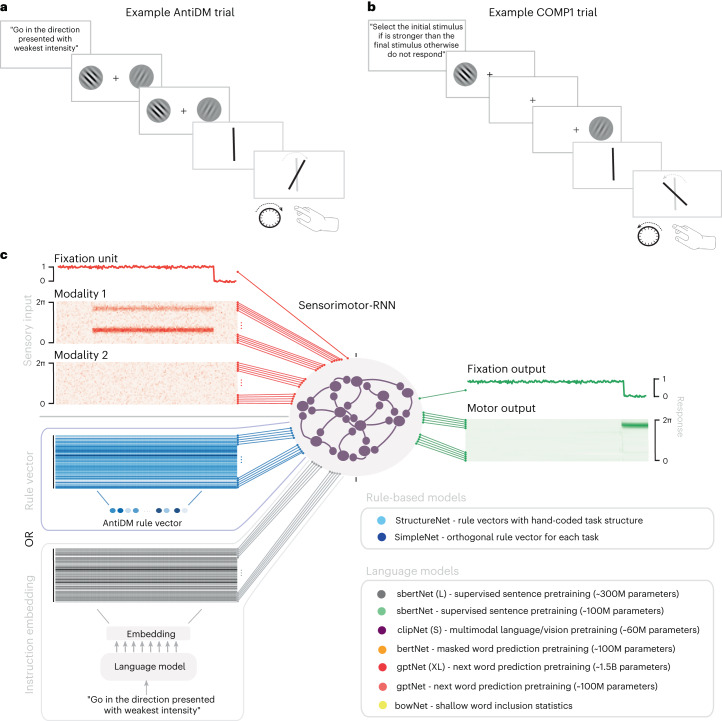
Tasks and models. **a**,**b**, Illustrations of example trials as they might appear in a laboratory setting. The trial is instructed, then stimuli are presented with different angles and strengths of contrast. The agent must then respond with the proper angle during the response period. **a**, An example AntiDM trial where the agent must respond to the angle presented with the least intensity. **b**, An example COMP1 trial where the agent must respond to the first angle if it is presented with higher intensity than the second angle otherwise repress response. **c**, Diagram of model inputs and outputs. Sensory inputs (fixation unit, modality 1, modality 2) are shown in red and model outputs (fixation output, motor output) are shown in green. Models also receive a rule vector (blue) or the embedding that results from passing task instructions through a pretrained language model (gray). A list of models tested is provided in the inset.

We train sensorimotor-RNNs on a set of 50 interrelated psychophysical tasks that require various cognitive capacities that are well studied in the literature^18^. Two example tasks are presented in Fig. 1a,b as they might appear in a laboratory setting. For all tasks, models receive a sensory input and task-identifying information and must output motor response activity (Fig. 1c). Input stimuli are encoded by two one-dimensional maps of neurons, each representing a different input modality, with periodic Gaussian tuning curves to angles (over (0, 2*π*)). Output responses are encoded in the same way. Inputs also include a single fixation unit. After the input fixation is off, the model can respond to the input stimuli. Our 50 tasks are roughly divided into 5 groups, ‘Go’, ‘Decision-making’, ‘Comparison’, ‘Duration’ And ‘Matching’, where within-group tasks share similar sensory input structures but may require divergent responses. For instance, in the decision-making (DM) task, the network must respond in the direction of the stimulus with the highest contrast, whereas in the anti-decision-making (AntiDM) task, the network responds to the stimulus with the weakest contrast (Fig. 1a). Thus, networks must properly infer the task demands for a given trial from task-identifying information in order to perform all tasks simultaneously (see Methods for task details; see Supplementary Fig. 13 for example trials of all tasks).

In our models, task-identifying input is either nonlinguistic or linguistic. We use two nonlinguistic control models. First, in SIMPLENET, the identity of a task is represented by one of 50 orthogonal rule vectors. Second, STRUCTURENET uses a set of 10 orthogonal structure vectors, each representing a dimension of the task set (that is, respond weakest versus strongest direction), and tasks are encoded using combinations of these vectors (see Supplementary Notes 3 for the full set of structure combinations). As a result, STRUCTURENET fully captures all the relevant relationships among tasks, whereas SIMPLENET encodes none of this structure.

Instructed models use a pretrained transformer architecture^19^ to embed natural language instructions for the tasks at hand. For each task, there is a corresponding set of 20 unique instructions (15 training, 5 validation; see Supplementary Notes 2 for the full instruction set). We test various types of language models that share the same basic architecture but differ in their size and also their pretraining objective. We tested two autoregressive models, a standard and a large version of GPT2, which we call GPT and GPT (XL), respectively. Previous work has demonstrated that GPT activations can account for various neural signatures of reading and listening^11^. BERT is trained to identify masked words within a piece of text^20^, but it also uses an unsupervised sentence-level objective, in which the network is given two sentences and must determine whether they follow each other in the original text. SBERT is trained like BERT but receives additional tuning on the Stanford Natural Language Inference task, a hand-labeled dataset detailing the logical relationship between two candidate sentences (Methods)^21,22^. Lastly, we use the language embedder from CLIP, a multimodal model that learns a joint embedding space of images and text captions^23^. We call a sensorimotor-RNN using a given language model LANGUAGEMODELNET and append a letter indicating its size. The various sizes of models are given in Fig. 1c. For each language model, we apply a pooling method to the last hidden state of the transformer and pass this fixed-length representation through a set of linear weights that are trained during task learning. This results in a 64-dimensional instruction embedding across all models (Methods). Language model weights are frozen unless otherwise specified. Finally, as a control, we also test a bag-of-words (BoW) embedding scheme that only uses word count statistics to embed each instruction.

First, we verify our models can perform all tasks simultaneously. For instructed models to perform well, they must infer the common semantic content between 15 distinct instruction formulations for each task. We find that all our instructed models can learn all tasks simultaneously except for GPTNET, where performance asymptotes are below the 95% threshold for some tasks. Hence, we relax the performance threshold to 85% for models that use GPT (Supplementary Fig. 1; see Methods for training details). We additionally tested all architectures on validation instructions (Supplementary Fig. 2). SBERTNET (L) and SBERTNET are our best-performing models, achieving an average performance of 97% and 94%, respectively, on validation instructions, demonstrating that these networks infer the proper semantic content even for entirely novel instructions.

### Generalization to novel tasks

**Fig. 2:**
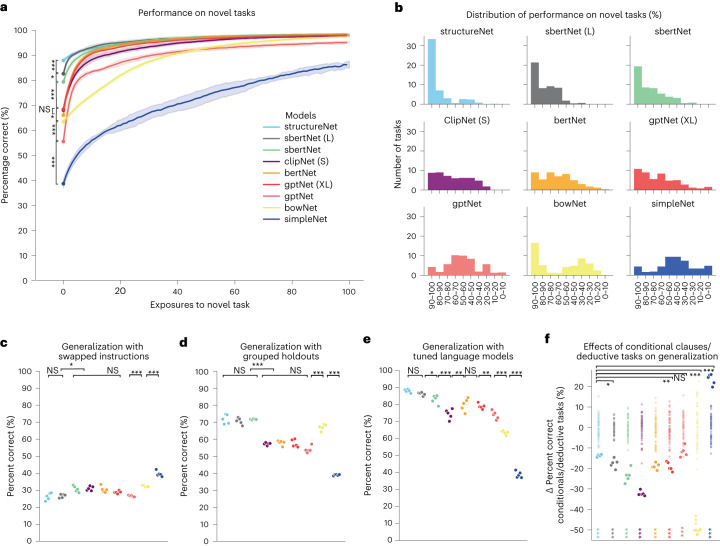
Model performance on novel tasks. **a**, Learning curves for the first 100 exposures to held-out tasks averaged over all tasks. Data are presented as the mean ± s.d. across different *n* = 5 random initializations of sensorimotor-RNN weights. For all subplots, asterisks indicate significant differences among performance according to a two-sided unequal-variance *t*-test. Most relevant comparisons are presented in plots (for all subplots, not significant (NS), *P* > 0.05, *P < 0.05, **P < 0.01, ***P < 0.001; STRUCTURENET versus SBERTNET (L): *t* = 3.761, *P* = 1.89 × 10^−4^; SBERTNET (L) versus SBERTNET: *t* = 2.19, *P* = 0.029; SBERTNET versus CLIPNET: *t* = 6.22, *P* = 1.02 × 10^−9^; CLIPNET versus BERTNET: *t* = 1.037, *P* = 0.300; BERTNET versus GPTNET (XL): *t* = −1.122, *P* = 0.262; GPTNET (XL) versus GPTNET: *t* = 6.22, *P* = 1.04 × 10^−9^; GPTNET versus BOWNET: *t* = −3.346, *P* = 8.85 × 10^−^^4^; BOWNET versus SIMPLENET: *t* = 10.25, *P* = 2.091 × 10^−22^). A full table of pairwise comparisons can be found in Supplementary Fig. 3. **b**, Distribution of generalization performance (that is, first exposure to novel task) across models. **c**–**f**, Performance across different test conditions for *n* = 5 different random initialization of sensorimotor-RNN weights where each point indicates average performance across tasks for a given initialization. **c**, Generalization performance for tasks where instructions are swapped at test time (STRUCTURENET versus SBERTNET (L): *t* = −0.15, *P* = 0.875; SBERTNET (L) versus SBERTNET: *t* = −2.102, *P* = 0.036; SBERTNET versus CLIPNET: *t* = −0.162, *P* = 0.871; CLIPNET versus BERTNET: *t* = 0.315, *P* = 0.752; BERTNET versus GPTNET (XL): *t* = 0.781, *P* = 0.435; GPTNET (XL) versus GPTNET: *t* = 1.071, *P* = 0.285; GPTNET versus BOWNET: *t* = −2.702, *P* = 0.007; BOWNET versus SIMPLENET: *t* = −3.471, *P* = 5.633^−4^). A full table of pairwise comparisons can be found in Supplementary Fig. 4. **d**, Generalization performance for models where tasks from the same family are held out during training (STRUCTURENET versus SBERTNET (L): *t* = 0.629, *P* = 0.530; SBERTNET (L) versus SBERTNET: *t* = −0.668, *P* = 0.504; SBERTNET versus CLIPNET: *t* = 8.043, *P* = 7.757 × 10^−15^; CLIPNET versus BERTNET: *t* = −0.306, *P* = 0.759; BERTNET versus GPTNET (XL): *t* = 0.163, *P* = 0.869; GPTNET (XL) versus GPTNET: *t* = 1.534, *P* = 0.126; GPTNET versus BOWNET: *t* = −6.418, *P* = 3.26 × 10^−10^; BOWNET versus SIMPLENET: *t* = 14.23, *P* = 8.561^−39^). A full table of pairwise comparisons can be found in Supplementary Fig. 4. **e**, Generalization performance for models where the last layers of language models are allowed to fine-tune to the loss from sensorimotor tasks (STRUCTURENET versus SBERTNET (L): *t* = 1.203, *P* = 0.229; SBERTNET (L) versus SBERTNET: *t* = 2.399, *P* = 0.016; SBERTNET versus CLIPNET: *t* = 5.186, *P* = 3.251 × 10^−7^; CLIPNET versus BERTNET: *t* = −3.002, *P* = 0.002; BERTNET versus GPTNET (XL): *t* = 0.522, *P* = 0.601; GPTNET (XL) versus GPTNET: *t* = 2.631, *P* = 0.009; GPTNET versus BOWNET: *t* = 4.440, *P* = 1.134 × 10^−5^; BOWNET versus SIMPLENET: *t* = 10.255, *P* = 2.091 × 10^−22^). A full table of pairwise comparisons can be found in Supplementary Fig. 4. **f**, Average difference in performance between tasks that use standard imperative instructions and those that use instructions with conditional clauses and require a simple deductive reasoning component. Colored asterisks at the bottom of the plot show *P* values for a two-sided, unequal-variance *t*-test between the null distribution constructed using random splits of the task set (transparent points represent mean differences for random splits; STRUCTURENET: *t* = −36.46, *P* = 4.34 × 10^−23^; SBERTNET (L): *t* = −16.38, *P* = 3.02 × 10^−5^; SBERTNET: *t* = −15.35, *P* = 3.920 × 10^−5^; CLIPNET: *t* = −44.68, *P* = 5.32 × 10^−^^13^; BERTNET: *t* = −25.51, *P* = 3.14 × 10^−8^; GPTNET (XL): *t* = −16.99, *P* = 3.61 × 10^−6^; GPTNET: *t* = −9.150, *P* = 0.0002; BOWNET: *t* = −70.99, *P* = 4.566 × 10^−35^; SIMPLENET: *t* = 19.60, *P* = 5.82 × 10^−6^), and asterisks at the top of plot indicate *P*-value results from a *t*-test comparing differences with STRUCTURENET and our other instructed models (versus SBERTNET (L): *t* = 3.702, *P* = 0.0168; versus SBERTNET: *t* = 6.592, *P* = 0.002; versus CLIPNET: *t* = 30.35, *P* = 2.367 × 10^−7^; versus BERTNET: *t* = 7.234, *P* = 0.0007; versus GPTNET (XL): *t* = 5.282, *P* = 0.004; versus GPTNET: *t* = −1.745, *P* = 0.149; versus BOWNET: *t* = 75.04, *P* = 9.96 × 10^−11^; versus SIMPLENET: *t* = −30.95, *P* = 2.86 × 10^−^^6^; see Methods and Supplementary Fig. 6. for full comparisons).

We next examined the extent to which different language models aided generalization to novel tasks. We trained individual networks on 45 tasks and then tested performance when exposed to the five held-out tasks. We use unequal-variance *t*-tests to make comparisons among the performance of different models. Figure 2 shows results with *P* values for the most relevant comparisons (a full matrix of comparisons across all models can be found in Supplementary Figs. 3 and 4)

Our uninstructed control model SIMPLENET performs at 39%, on average, on the first presentation of a novel task (zero-shot generalization). This serves as a baseline for generalization. Note that despite the orthogonality of task rules provided to SIMPLENET, exposure to the task set allows models to learn patterns that are common to all tasks (for example, always repress response during fixation). Therefore, 39% is not chance-level performance per se, but rather performance achieved by a network trained and tested on a task set with some common requirements for responding. GPTNET, exhibits a zero-shot generalization of 57%. This is a significant improvement over SIMPLENET (*t* = 8.32, *P* = 8.24 × 10^−16^). Strikingly, increasing the size of GPT by an order of magnitude to the 1.5 billion parameters used by GPT (XL) only resulted in modest gains over BOWNET (64%), with GPTNET (XL) achieving 68% on held-out tasks (*t* = 2.04, *P* = 0.047). By contrast, CLIPNET (S), which uses 4% of the number of parameters utilized by GPTNET (XL), is nonetheless able to achieve the same performance (68% correct, *t* = 0.146, *P* = 0.88). Likewise, BERTNET achieves a generalization performance that lags only 2% behind GPTNETXL in the mean (*t* = −1.122, *P* = 0.262). By contrast, models with knowledge of sentence-level semantics show marked improvements in generalization, with SBERTNET performing an unseen task at 79% correct on average. Finally, our best-performing model, SBERTNET (L), can execute a never-before-seen task with a performance of 83% correct, on average, lagging just a few percentage points behind STRUCTURENET (88% correct), which receives the structure of the task set hand-coded in its rule vectors.

Figure 2b shows a histogram of the number of tasks for which each model achieves a given level of performance. Again, SBERTNET (L) manages to perform over 20 tasks set nearly perfectly in the zero-shot setting (for individual task performance for all models across tasks, see Supplementary Fig. 3).

To validate that our best-performing models leveraged the semantics of instructions, we presented the sensory input for one held-out task while providing the linguistic instructions for a different held-out task. Models that truly rely on linguistic information should be most penalized by this manipulation and, as predicted, we saw the largest decrease in performance for our best models (Fig. 2c).

We also tested a more stringent hold-out procedure where we purposefully chose 4–6 tasks from the same family of tasks to hold out during training (Fig. 2d). Overall, performance decreased in this more difficult setting, although our best-performing models still showed strong generalization, with SBERTNET (L) and SBERTNET achieving 71% and 72% correct on novel tasks, respectively, which was not significantly different from STRUCTURENET at 72% (*t* = 0.629, *P* = 0.529; *t* = 0.064, *P* = 0.948; for SBERTNET (L) and SBERTNET, respectively).

In addition, we tested models in a setting where we allow the weights of language models to tune according to the loss experienced during sensorimotor training (see Methods for tuning details). This manipulation improved the generalization performance across all models, and for our best-performing model, SBERTNET (L), we see that generalization is as strong as for STRUCTURENET (86%, *t* = 1.204, *P* = 0.229).

Following ref. ^18^, we tested models in a setting where task-type information for a given task was represented as a composition of information for related tasks in the training set (that is, AntiDMMod1 = (rule(AntiDMMod2) − rule(DMMod2)) + rule(DMMod1)). In this setting, we did find that the performance of SIMPLENET improved (60% correct). However, when we combined embedded instructions according to the same compositional rules, our linguistic models dramatically outperformed SIMPLENET. This suggests that training in the context of language more readily allows a simple compositional scheme to successfully configure task responses (see Supplementary Fig. 5 for full results and compositional encodings).

Finally, we tested a version of each model where outputs of language models are passed through a set of nonlinear layers, as opposed to the linear mapping used in the preceding results. We found that this manipulation reduced performance, suggesting that this added power leads to overfitting on training tasks, and that a simpler linear mapping is better suited to generalization (see Methods for details and Supplementary Fig. 4 for full results).

The discrepancy in performance between our instructed models suggests that in order to represent linguistic information such that it can successfully configure sensorimotor networks, it is not sufficient to simply use any very powerful language processing system. Rather, model success can be delineated by the extent to which they are exposed to sentence-level semantics during pretraining. Our best-performing models SBERTNET (L) and SBERTNET are explicitly trained to produce good sentence embeddings, whereas our worst-performing model, GPTNET, is only tuned to the statistics of upcoming words. Both CLIPNET (S) and BERTNET are exposed to some form of sentence-level knowledge. CLIPNET (S) is interested in sentence-level representations, but trains these representations using the statistics of corresponding vision representations. BERTNET performs a two-way classification of whether or not input sentences are adjacent in the training corpus. That the 1.5 billion parameters of GPTNET (XL) doesn’t markedly improve performance relative to these comparatively small models speaks to the fact that model size isn’t the determining factor. Lastly, although BoW removes key elements of linguistic meaning (that is, syntax), the simple use of word occurrences encodes information primarily about the similarities and differences between the sentences. For instance, simply representing the inclusion or exclusion of the words ‘stronger’ or ‘weaker’ is highly informative about the meaning of the instruction.

We also investigated which features of language make it difficult for our models to generalize. Thirty of our tasks require processing instructions with a conditional clause structure (for example, COMP1) as opposed to a simple imperative (for example, AntiDM). Tasks that are instructed using conditional clauses also require a simple form of deductive reasoning (if *p* then *q* else *s*). Neuroimaging literature exploring the relationship between such deductive processes and language areas has reached differing conclusions, with some early studies showing that deduction recruits regions that are thought to support syntactic computations^24–26^ and follow-up studies claiming that deduction can be reliably dissociated from language areas^27–30^. One theory for this variation in results is that baseline tasks used to isolate deductive reasoning in earlier studies used linguistic stimuli that required only superficial processing^31,32^.

To explore this issue, we calculated the average difference in performance between tasks with and without conditional clauses/deductive reasoning requirements (Fig. 2f). All our models performed worse on these tasks relative to a set of random shuffles. However, we also saw an additional effect between STRUCTURENET and our instructed models, which performed worse than STRUCTURENET by a statistically significant margin (see Supplementary Fig. 6 for full comparisons). This is a crucial comparison because STRUCTURENET performs deductive tasks without relying on language. Hence, the decrease in performance between STRUCTURENET and instructed models is in part due to the difficulty inherent in parsing syntactically more complicated language. The implication is that we may see engagement of linguistic areas in deductive reasoning tasks, but this may simply be due to the increased syntactic demands of corresponding instructions (rather than processes that recruit linguistic areas to explicitly aid in the deduction). This result largely agrees with two reviews of the deductive reasoning literature, which concluded that the effects in language areas seen in early studies were likely due to the syntactic complexity of test stimuli^31,32^.

### Shared structure in language and sensorimotor networks

**Fig. 3:**
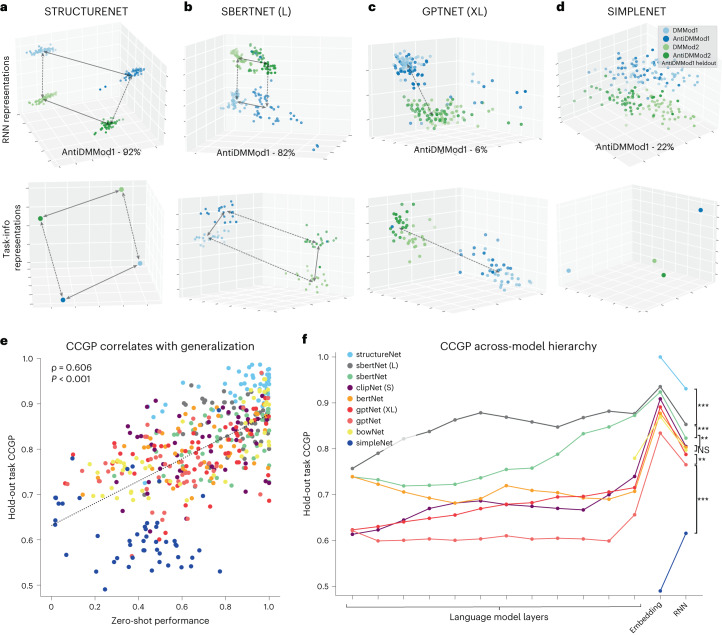
Structured representations in instructed models. **a**–**d**, The first three PCs of sensorimotor hidden activity and task-info representations for models trained with AntiDMMod1 held out. Solid arrows represent an abstract ‘Pro’ versus ‘Anti’ axis, and dashed arrows represent an abstract ‘Mod1’ versus ‘Mod2’ axis. **a**, STRUCTURENET. **b**, SBERTNET (L). **c**, GPTNET (XL). **d**, SIMPLENET. **e**, Correlation between held-out task CCGP and zero-shot performance (Pearson’s *r* = 0.606, *P* = 1.57 × 10^−46^). **f**, CCGP scores for held-out tasks for each layer in the model hierarchy. Significance scores indicate *P-*value results from pairwise two-sided unequal-variance *t*-tests performed among model distributions of CCGP scores on held-out tasks for sensorimotor-RNN (NS *P* > 0.05, *P < 0.05, **P < 0.01, ***P < 0.001; STRUCTURENET versus SBERTNET (L): *t* = 13.67, *P* = 2.44 × 10^−36^; SBERTNET (L) versus SBERTNET: *t* = 5.061, *P* = 5.84 × 10^−7^; SBERTNET versus CLIPNET: *t* = 2.809, *P* = 0.005; CLIPNET versus BERTNET: *t* = 0.278, *P* = 0.780; BERTNET versus GPTNET (XL): *t* = 2.505, *P* = 0.012; GPTNET (XL) versus GPTNET: *t* = 3.180, *P* = 0.001; GPTNET versus BOWNET: *t* = −4.176, *P* = 3.50 × 10^−5^; BOWNET versus SIMPLENET: *t* = 23.0.8, *P* = 1.10^−80^; see Supplementary Fig. 9 for full comparisons as well as *t*-test results for embedding layer CCGP scores).

We then turned to an investigation of the representational scheme that supports generalization. First, we note that like in other multitasking models, units in our sensorimotor-RNNs exhibited functional clustering, where similar subsets of neurons show high variance across similar sets of tasks (Supplementary Fig. 7). Moreover, we found that models can learn unseen tasks by only training sensorimotor-RNN input weights and keeping the recurrent dynamics constant (Supplementary Fig. 8). Past work has shown that these properties are characteristic of networks that can reuse the same set of underlying neural resources across different settings^6,18^. We then examined the geometry that exists between the neural representations of related tasks. We plotted the first three principal components (PCs) of sensorimotor-RNN hidden activity at stimulus onset in SIMPLENET, GPTNETXL, SBERTNET (L) and STRUCTURENET performing modality-specific DM and AntiDM tasks. Here, models receive input for a decision-making task in both modalities but must only attend to the stimuli in the modality relevant for the current task. Importantly, AntiDMMod1 is held out of training in the following examples. In addition, we plotted the PCs of either the rule vectors or the instruction embeddings in each task (Fig. 3).

For STRUCTURENET, hidden activity is factorized along task-relevant axes, namely a consistent ‘Pro’ versus ‘Anti’ direction in activity space (solid arrows), and a ‘Mod1’ versus ‘Mod2’ direction (dashed arrows). Importantly, this structure is maintained even for AntiDMMod1, which has been held out of training, allowing STRUCTURENET to achieve a performance of 92% correct on this unseen task. This factorization is also reflected in the PCs of rule embeddings. Strikingly, SBERTNET (L) also organizes its representations in a way that captures the essential compositional nature of the task set using only the structure that it has inferred from the semantics of instructions. This is the case for language embeddings, which maintain abstract axes across AntiDMMod1 instructions (again, held out of training). As a result, SBERTNET (L) is able to use these relevant axes for AntiDMMod1 sensorimotor-RNN representations, leading to a generalization performance of 82%. By contrast, GPTNET (XL) fails to properly infer a distinct ‘Pro’ versus ‘Anti’ axes in either sensorimotor-RNN representations or language embeddings leading to a zero-shot performance of 6% on AntiDMMod1 (Fig. 3b). Finally, we find that the orthogonal rule vectors used by simpleNet preclude any structure between practiced and held-out tasks, resulting in a performance of 22%.

To more precisely quantify this structure, we measure the cross-conditional generalization performance (CCGP) of these representations^3^. CCGP measures the ability of a linear decoder trained to differentiate one set of conditions (that is, DMMod2 and AntiDMMod2) to generalize to an analogous set of test conditions (that is, DMMod1 and AntiDMMod1). Intuitively, this captures the extent to which models have learned to place sensorimotor activity along abstract task axes (that is, the ‘Anti’ dimension). Notably, high CCGP scores and related measures have been observed in experiments that required human participants to flexibly switch between different interrelated tasks^4,33^.

We measured CCGP scores among representations in sensorimotor-RNNs for tasks that have been held out of training (Methods) and found a strong correlation between CCGP scores and zero-shot performance (Fig. 3e). Additionally, we find that swapping task instructions for held-out tasks dramatically reduces CCGP scores for all our instructed models, indicating that the semantic of instructions is crucial for maintaining structured representations (Supplementary Fig. 9).

We then looked at how structure emerges in the language processing hierarchy. CCGP decoding scores for different layers in our model are shown in Fig. 3f. For each instructed model, scores for 12 transformer layers (or the last 12 layers for SBERTNET (L) and GPTNET (XL)), the 64-dimensional embedding layer and the Sensorimotor-RNN task representations are plotted. We also plotted CCGP scores for the rule embeddings used in our nonlinguistic models. Among models, there was a notable discrepancy in how abstract structure emerges. Autoregressive models (GPTNETXL, GPTNET), BERTNET and CLIPNET (S), showed a low CCGP throughout language model layers followed by a jump in the embedding layer. This is because weights feeding into the embedding layer are tuned during sensorimotor training. The implication of this spike is that most of the useful representational processing in these models actually does not occur in the pretrained language model per se, but rather in the linear readout, which is exposed to task structure via training. By contrast, our best-performing models SBERTNET and SBERTNET (L) use language representations where high CCGP scores emerge gradually in the intermediate layers of their respective language models. Because semantic representations already have such a structure, most of the compositional inference involved in generalization can occur in the comparatively powerful language processing hierarchy. As a result, representations are already well organized in the last layer of language models, and a linear readout in the embedding layer is sufficient for the sensorimotor-RNN to correctly infer the geometry of the task set and generalize well.

This analysis strongly suggests that models exhibiting generalization do so by leveraging structured semantic representations to properly relate practiced and novel tasks in sensorimotor space, thereby allowing a composition of practiced behaviors in an unseen setting.

### Semantic modulation of single-unit tuning properties

Next, we examined tuning profiles of individual units in our sensorimotor-RNNs. We found that individual neurons are tuned to a variety of task-relevant variables. Critically, however, we find neurons where this tuning varies predictably within a task group and is modulated by the semantic content of instructions in a way that reflects task demands.

**Fig. 4:**
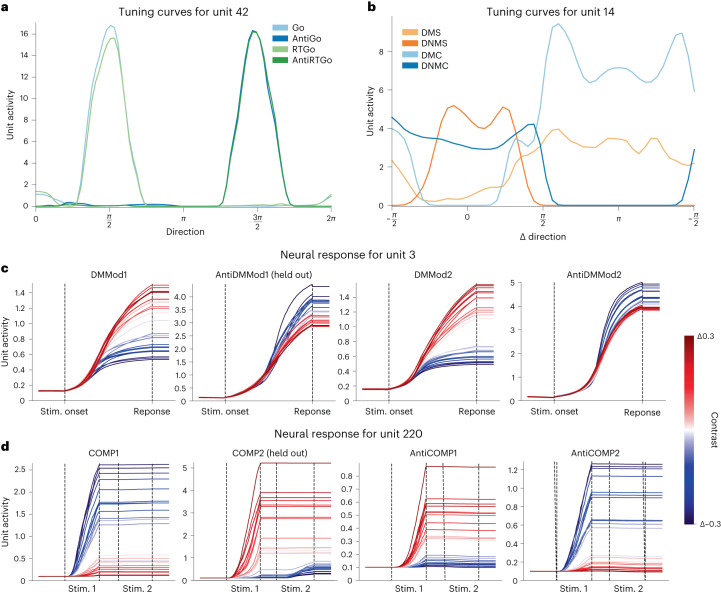
Semantic modulation of single-unit tuning properties. **a**, Tuning curves for a SBERTNET (L) sensorimotor-RNN unit that modulates tuning according to task demands in the ‘Go’ family. **b**, Tuning curves, for a SBERTNET (L) sensorimotor-RNN unit in the ‘matching’ family of tasks plotted in terms of difference in angle between two stimuli. **c**, Full activity traces for modality-specific ‘DM’ and ‘AntiDM’ tasks for different levels of relative stimulus strength. **d**, Full activity traces for tasks in the ‘comparison’ family of tasks for different levels of relative stimulus strength.

For instance, in the ‘Go’ family of tasks, unit 42 shows direction selectivity that shifts by *π* between ‘Pro’ and ‘Anti’ tasks, reflecting the relationship of task demands in each context (Fig. 4a). This flip in selectivity is observed even for the AntiGo task, which was held out during training.

For the ‘Matching’ family of tasks, unit 14 modulates activity between ‘match’ (DMS, DMC) and ‘non-match’ (DNMS, DNMC) conditions. In ‘non-match’ trials, the activity of this unit increases as the distance between the two stimuli increases. By contrast, for ‘matching’ tasks, this neuron is most active when the relative distance between the two stimuli is small. Hence, in both cases this neuron modulates its activity to represent when the model should respond, changing selectivity to reflect opposing task demands between ‘match’ and ‘non-match’ trials. This is true even for DMS, which has been held out of training.

Figure 4c shows traces of unit 3 activity in modality-specific versions of DM and AntiDM tasks (AntiDMMod1 is held out of training) for different levels of contrast (contrast = *str*_stim1_ − *str*_stim2_). In all tasks, we observed ramping activity where the rate of ramping is relative to the strength of contrast. This motif of activity has been reported in previous studies^34,35^. However, in our models, we observe that an evidence-accumulating neuron can swap the sign of its integration in response to a change in linguistic instructions, which allows models to meet opposing demands of ‘Pro’ and ‘Anti’ versions of the task, even for previously unseen tasks.

Interestingly, we also found that unsuccessful models failed to properly modulate tuning preferences. For example, with GPTNET (XL), which failed to factorize along a ‘Pro’ versus ‘Anti’ axis (Fig. 3b) and had poor generalization on AntiDMMod1, we also find neurons that failed to swap their sign of integration in the held-out setting (Supplementary Fig. 10).

Finally, we see a similar pattern in the time course of activity for trials in the ‘Comparison’ family of tasks (Fig. 4d). In the COMP1 task, the network must respond in the direction of the first stimulus if it has higher intensity than the second stimulus, and must not respond otherwise. In COMP2, it must only respond to the second stimulus if the second stimulus is higher intensity. For ‘Anti’ versions, the demands of stimulus ordering are the same except the model has to choose the stimuli with the weakest contrast. Even with this added complexity, we found individual neurons that modulate their tuning with respect to task demands, even for held-out tasks (in this case COMP2). For example, unit 82 is active when the network should repress response. For ‘COMP1’, this unit is highly active with negative contrast (that is, *str*_stim2_ > *str*_stim1_), but flips this sensitivity in COMP2 and is highly active with positive contrast (that is, *str*_stim1_ > *str*_stim2_). Importantly, this relation is reversed when the goal is to select the weakest stimuli. Hence, despite these subtle syntactic differences in instruction sets, the language embedding can reverse the tuning of this unit in a task-appropriate manner.

### Linguistic communication between networks

**Fig. 5:**
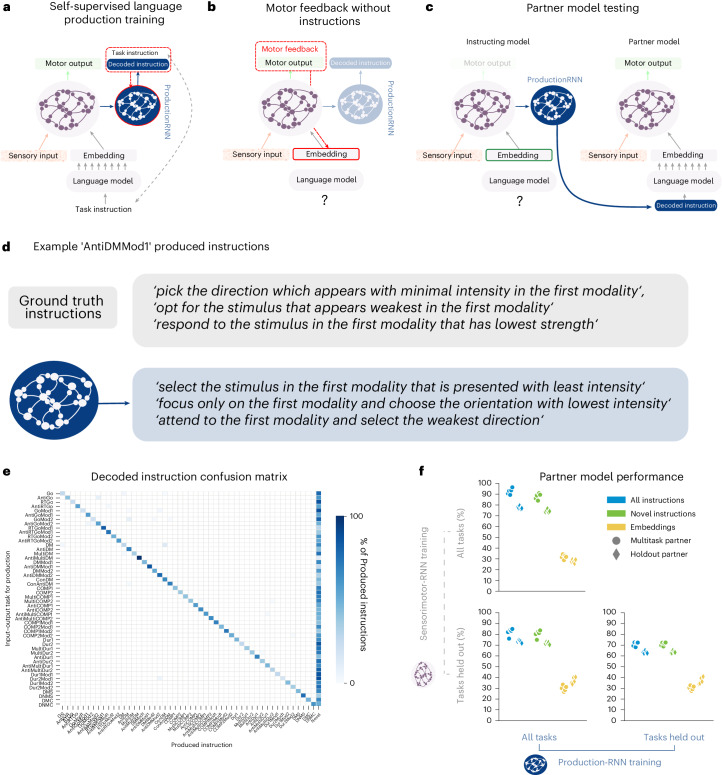
Communication between networks. **a**, Illustration of self-supervised training procedure for the language production network (blue). The red dashed line indicates gradient flow. **b**, Illustration of motor feedback used to drive task performance in the absence of linguistic instructions. **c**, Illustration of the partner model evaluation procedure used to evaluate the quality of instructions generated from the instructing model. **d**, Three example instructions produced from sensorimotor activity evoked by embeddings inferred in **b** for an AntiDMMod1 task. **e**, Confusion matrix of instructions produced again using the method described in **b**. *y* axis indicates input–output task used to infer an embedding, and *x* axis indicates whether the instruction produced from the resulting sensorimotor activity was included in one of the instruction sets used during self-supervised training or else was a ‘novel’ formulation. **f**, Performance of partner models in different training regimes given produced instructions or direct input of embedding vectors. Each point represents the average performance of a partner model across tasks using instructions from decoders train with different random initializations. Dots indicate the partner model was trained on all tasks, whereas diamonds indicate performance on held-out tasks. Axes indicate the training regime of the instructing model. Full statistical comparisons of performance can be found in Supplementary Fig. 12.

We now seek to model the complementary human ability to describe a particular sensorimotor skill with words once it has been acquired. To do this, we inverted the language-to-sensorimotor mapping our models learn during training so that they can provide a linguistic description of a task based only on the state of sensorimotor units. First, we constructed an output channel (production-RNN; Fig. 5a–c), which is trained to map sensorimotor-RNN states to input instructions. We then present the network with a series of example trials while withholding instructions for a specific task. During this phase all model weights are frozen, and models receive motor feedback in order to update the embedding layer activity in order to reduce the error of the output (Fig. 5b). Once the activity in the embedding layer drives sensorimotor units to achieve a performance criterion, we used the production-RNN to decode a linguistic description of the current task. Finally, to evaluate the quality of these instructions, we input them into a partner model and measure performance across tasks (Fig. 5c). All instructing and partner models used in this section are instances of SBERTNET (L) (Methods).

Some example decoded instructions for the AntiDMMod1 task (Fig. 5d; see Supplementary Notes 4 for all decoded instructions). To visualize decoded instructions across the task set, we plotted a confusion matrix where both sensorimotor-RNN and production-RNN are trained on all tasks (Fig. 5e). Note that many decoded instructions were entirely ‘novel’, that is, they were not included in the training set for the production-RNN (Methods). Novel instructions made up 53% of decoded instructions across all tasks.

To test the quality of these novel instructions, we evaluated a partner model’s performance on instructions generated by the first network (Fig. 5c; results are shown in Fig. 5f). When the partner model is trained on all tasks, performance on all decoded instructions was 93% on average across tasks. Communicating instructions to partner models with tasks held out of training also resulted in good performance (78%). Importantly, performance was maintained even for ‘novel’ instructions, where average performance was 88% for partner models trained on all tasks and 75% for partner models with hold-out tasks. Given that the instructing and partner models share the same architecture, one might expect that it is more efficient to forgo the language component of communication and simply copy the embedding inferred by one model into the input of the partner model. This resulted in only 31% correct performance on average and 28% performance when testing partner models on held-out tasks. Although both instructing and partner networks share the same architecture and the same competencies, they nonetheless have different synaptic weights. Hence, using a neural representation tuned for the set of weights within the one agent won’t necessarily produce good performance in the other.

We also tested an instructing model using a sensorimotor-RNN with tasks held out of training. We emphasize here that during training the production-RNN attempts to decode from sensorimotor hidden states induced by instructions for tasks the network has never experienced before (Fig. 5a), whereas during test time, instructions are produced from sensorimotor states that emerge entirely as a result of minimizing a motor error (Fig. 5b,c). We nonetheless find that, in this setting, a partner model trained on all tasks performs at 82% correct, while partner models with tasks held out of training perform at 73%. Here, 77% of produced instructions are novel, so we see a very small decrease of 1% when we test the same partner models only on novel instructions. Like above, context representations induce a relatively low performance of 30% and 37% correct for partners trained on all tasks and with tasks held out, respectively.

Lastly, we tested our most extreme setting where tasks have been held out for both sensorimotor-RNNs and production-RNNs (Fig. 5f). We find that produced instructions induce a performance of 71% and 63% for partner models trained on all tasks and with tasks held out, respectively. Although this is a decrease in performance from our previous set-ups, the fact that models can produce sensible instructions at all in this double held-out setting is striking. The fact that the system succeeds to any extent speaks to strong inductive biases introduced by training in the context of rich, compositionally structured semantic representations.

## Discussion

In this study, we use the latest advances in natural language processing to build tractable models of the ability to interpret instructions to guide actions in novel settings and the ability to produce a description of a task once it has been learned. RNNs can learn to perform a set of psychophysical tasks simultaneously using a pretrained language transformer to embed a natural language instruction for the current task. Our best-performing models can leverage these embeddings to perform a brand-new model with an average performance of 83% correct. Instructed models that generalize performance do so by leveraging the shared compositional structure of instruction embeddings and task representations, such that an inference about the relations between practiced and novel instructions leads to a good inference about what sensorimotor transformation is required for the unseen task. Finally, we show a network can invert this information and provide a linguistic description for a task based only on the sensorimotor contingency it observes.

Our models make several predictions for what neural representations to expect in brain areas that integrate linguistic information in order to exert control over sensorimotor areas. Firstly, the CCGP analysis of our model hierarchy suggests that when humans must generalize across (or switch between) a set of related tasks based on instructions, the neural geometry observed among sensorimotor mappings should also be present in semantic representations of instructions. This prediction is well grounded in the existing experimental literature where multiple studies have observed the type of abstract structure we find in our sensorimotor-RNNs also exists in sensorimotor areas of biological brains^3,36,37^. Our models theorize that the emergence of an equivalent task-related structure in language areas is essential to instructed action in humans. One intriguing candidate for an area that may support such representations is the language selective subregion of the left inferior frontal gyrus. This area is sensitive to both lexico-semantic and syntactic aspects of sentence comprehension, is implicated in tasks that require semantic control and lies anatomically adjacent to another functional subregion of the left inferior frontal gyrus, which is implicated in flexible cognition^38–41^. We also predict that individual units involved in implementing sensorimotor mappings should modulate their tuning properties on a trial-by-trial basis according to the semantics of the input instructions, and that failure to modulate tuning in the expected way should lead to poor generalization. This prediction may be especially useful to interpret multiunit recordings in humans. Finally, given that grounding linguistic knowledge in the sensorimotor demands of the task set improved performance across models (Fig. 2e), we predict that during learning the highest level of the language processing hierarchy should likewise be shaped by the embodied processes that accompany linguistic inputs, for example, motor planning or affordance evaluation^42^.

One notable negative result of our study is the relatively poor generalization performance of GPTNET (XL), which used at least an order of magnitude more parameters than other models. This is particularly striking given that activity in these models is predictive of many behavioral and neural signatures of human language processing^10,11^. Given this, future imaging studies may be guided by the representations in both autoregressive models and our best-performing models to delineate a full gradient of brain areas involved in each stage of instruction following, from low-level next-word prediction to higher-level structured-sentence representations to the sensorimotor control that language informs.

Our models may guide future work comparing compositional representations in nonlinguistic subjects like nonhuman primates. Comparison of task switching (without linguistic instructions) between humans and nonhuman primates indicates that both use abstract rule representations, although humans can make switches much more rapidly^43^. One intriguing parallel in our analyses is the use of compositional rules vectors (Supplementary Fig. 5). Even in the case of nonlinguistic SIMPLENET, using these vectors boosted generalization. Importantly, however, this compositionality is much stronger for our best-performing instructed models. This suggests that language endows agents with a more flexible organization of task subcomponents, which can be recombined in a broader variety of contexts.

Our results also highlight the advantages of linguistic communication. Networks can compress the information they have gained through experience of motor feedback and transfer that knowledge to a partner network via natural language. Although rudimentary in our example, the ability to endogenously produce a description of how to accomplish a task after a period of practice is a hallmark human language skill. The failure to transfer performance by sharing latent representations demonstrates that to communicate information in a group of independent networks of neurons, it needs to pass through a representational medium that is equally interpretable by all members of the group. In humans and for our best-performing instructed models, this medium is language.

A series of works in reinforcement learning has investigated using language and language-like schemes to aid agent performance. Agents receive language information through step-by-step descriptions of action sequences^44,45^, or by learning policies conditioned on a language goal^46,47^. These studies often deviate from natural language and receive linguistic inputs that are parsed or simply refer directly to environmental objects. Some larger versions of the pretrained language models we use to embed instructions also display instructions following behavior, that is, GPT-3 (ref. ^7^), PALM^12^, LaMDA^13^ and InstructGPT^48^ in the modality of language and DALL-E^8^ and Stable Diffusion^14^ in a language to image modality. The semantic and syntactic understanding displayed in these models is impressive. However, the outputs of these models are difficult to interpret in terms of guiding the dynamics of a downstream action plan. Finally, recent work has sought to engineer instruction following agents that can function in complex or even real-world environments^16–18^. While these models exhibit impressive behavioral repertoires, they rely on perceptual systems that fuse linguistic and visual information making them difficult to compare to language representations in human brains, which emerge from a set of areas specialized for processing language. In all, none of these models offer a testable representational account of how language might be used to induce generalization over sensorimotor mappings in the brain.

Our models by contrast make tractable predictions for what population and single-unit neural representations are required to support compositional generalization and can guide future experimental work examining the interplay of linguistic and sensorimotor skills in humans. By developing interpretable models that can both understand instructions as guiding a particular sensorimotor response, and communicate the results of sensorimotor learning as an intelligible linguistic instruction, we have begun to explain the power of language in encoding and transferring knowledge in networks of neurons.

## Methods

### Model architecture

#### Sensorimotor-RNN

The base model architecture and task structure used in this paper follows^18^. All networks of sensorimotor units denoted sensorimotor-RNN are gated recurrent units (GRU)^49^ using rectified linear unit (ReLU) nonlinearities with 256 hidden units each. Inputs to the networks consist of (1) sensory inputs, *X*_*t*_ and (2) task-identifying information, *I*_*t*_. We initialize hidden activity in the GRU as ${h}^{0}\in {{\mathbb{R}}}^{256}$ with values set to 0.1. All networks of sensorimotor units use the same hidden state initialization, so we omit *h*^0^ in network equations. At each time step, a readout layer Linear_out_ decodes motor activity, $\hat{{y}_{t}}$, from the activity of recurrent hidden units, *h*_*t*_, according to:

$$\begin{array}{ll}{h}_{t}={{{\rm{SensorimotorRNN}}}}\Big({X}_{t},{I}_{t};{h}_{t-1}\Big)\qquad\qquad{h}_{t}\in {{\mathbb{R}}}^{256}\\ {\hat{y}}_{t}=\sigma \Big({{{{\rm{Linear}}}}}_{{{{\rm{out}}}}}({h}_{t})\Big)\qquad\qquad\qquad\qquad\qquad\quad{\hat{y}}_{t}\in {{\mathbb{R}}}^{33}\end{array}$$

where *σ* denotes the sigmoid function. Sensory inputs *X*_*t*_ are made up of three channels, two sensory modalities ${x}_{{{\mathrm{mod}}}\,1,t}$ and ${x}_{{{\mathrm{mod}}}\,2,t}$, and a fixation channel *x*_fix,*t*_. Both ${x}_{{{\mathrm{mod}}}\,1,t},{x}_{{{\mathrm{mod}}}\,2,t}\in {{\mathbb{R}}}^{32}$ and stimuli in these modalities are represented as hills of activity with peaks determined by units’ preferred directions around a one-dimensional circular variable. For an input at direction *θ*, the activity of a given input unit *u*_*i*_ with preferred direction *θ*_*i*_ is

$${u}_{i}=str \times 0.8\exp \left[-0.5 \times {\left(\frac{8| \theta -{\theta }_{i}| }{\pi }\right)}^{2}\right]$$

where *str* is the coefficient describing stimulus strength. The fixation channel ${x}_{{{{\rm{fix}}}},t}\in {{\mathbb{R}}}^{1}$ is a single unit simulating a fixation cue for the network. In all, sensory input ${X}_{t}=({x}_{mod1,t},{x}_{mod2,t},{x}_{fix,t})\in {{\mathbb{R}}}^{65}$. Motor output, ${\hat{{y}}_{t}}$ consists of both a 32-dimensional ring representing directional responses to the input stimulus as well as a single unit representing model fixation, so that ${\hat{{y}}_{t}}\in {{\mathbb{R}}}^{33}$.

For all models, task-identifying information ${I}_{t}\in {{\mathbb{R}}}^{64}$. Task-identifying information is presented throughout the duration of a trial and remains constant such that ${I}_{t}={I}_{t{\prime} }\forall t,t{\prime}$. For all models, task-identifying info *I*_*t*_ and sensory input *X*_*t*_ are concatenated as inputs to the sensorimotor-RNN.

#### Nonlinguistic models

For SIMPLENET, we generate a set of 64-dimensional orthogonal task rules by constructing an orthogonal matrix using the Python package scipy.stats.ortho_group, and assign rows of this matrix to each task type. For STRUCTURENET, we generate a set of ten orthogonal, 64-dimensional vectors in the same manner, and each of these represents a dimension of the task set (that is, respond weakest versus strongest direction, respond in the same versus opposite direction, pay attention only to stimuli in the first modality, and so on). Rule vectors for tasks are then simple combinations of each of these ten basis vectors. For a full description of structure rule vectors, see Supplementary Note 3.

We also test SIMPLENETPLUS and STRUCTURENETPLUS, which use an additional hidden layer with 128 units and ReLU nonlinearities to process orthogonal tasks rules *I*_*t*_ into a vector $\bar{{I}_{t}}$ which is used by sensorimotor-RNN as task-identifying information.

$$\begin{array}{ll}{\bar{{I}_{t}}}^{{\prime} }=\rm{ReLU}({{{{\rm{Linear}}}}}_{{{{\rm{RuleEmb}}}}1}({I}_{t}))&{\bar{{I}_{t}}}^{{\prime} }\in {{\mathbb{R}}}^{128}\\ {\bar{{I}_{t}}}^{{\prime} }=\rm{ReLU}({{{{\rm{Linear}}}}}_{{{{\rm{RuleEmb}}}}2}({I}_{t}^{{\prime} }))&{\bar{{I}_{t}}}^{{\prime} }\in {{\mathbb{R}}}^{128}\\ \bar{{I}_{t}}=\rm{ReLU}({{{{\rm{Linear}}}}}_{{{{\rm{RuleEmb}}}}3}({\bar{{I}_{t}}}^{{\prime} }))&\bar{{I}_{t}}\in {{\mathbb{R}}}^{64}\end{array}$$

Full results for these models are included in Supplementary Fig. 4.

#### Pretrained transformers

The main language models we test use pretrained transformer architectures to produce *I*. Importantly, transformers differ in the type of pretraining objective used to tune the model parameters. GPT is trained to predict the next word given a context of words^9^. GPT (XL) follows the same objective but trains for longer on a larger dataset^50^. Both models are fully autoregressive. BERT, by contrast, takes bidirectional language inputs and is tasked with predicting masked words that appear in the middle of input phrases. Additionally, BERT is trained on a simple sentence prediction task where the model must determine if input sentence 1 is followed by input sentence 2 in the training corpus. Extending this principle, SBERT is explicitly trained to produce fixed-length embeddings of whole sentences^21^. It takes pretrained BERT networks and uses them in a siamese architecture^51^, which allows the weights of the model to be tuned in a supervised fashion according to the Stanford Natural Language Inference dataset^22^. Natural language inference is a three-way categorization task where the network must infer the logical relationship between sentences: whether a premise sentence implies, contradicts or is unrelated to a hypothesis sentence. Finally, CLIP is trained to jointly embed images and language^23^. It uses data from captioned images and is asked to properly categorize which text and images pairs match or are mismatched in the dataset via a contrastive loss.

Importantly, the natural output of a transformer is a matrix of size ${\dim }_{{{{\rm{trans}}}}.}\times {{{\mathcal{T}}}}$, the inherent dimensionality of the transformer by the length of the input sequence. To create an embedding space for sentences it is standard practice to apply a pooling method to the transformer output, which produces a fixed-length representation for each instruction.

For GPT, GPT (XL), BERT and SBERT, we use an average pooling method. Suppose we have an input instruction ${w}_{1}\ldots {w}_{{{{\mathcal{T}}}}}$. Following standard practice with pretrained language models, the input to our transformers is tokenized with special ‘cls’ and ‘eos’ tokens at the beginning and end of the input sequence. We then compute *I* as follows:

$$\begin{array}{ll}{h}^{\rm{tran.}}={{{\rm{transformer}}}}\Big({{\mbox{[cls]}}}\,,{w}_{1}\ldots {w}_{{{{\mathcal{T}}}}},\,{{\mbox{[eos]}}}\Big),\qquad{h}^{\rm{tran.}}\in {{\mathbb{R}}}^{{\dim }_{{{{\rm{trans}}}}.}\times {{{\mathcal{T}}}}+2}\\ {h}^{I}={{{\rm{mean}}}}({h}^{\rm{tran.}}),\qquad\qquad\qquad\qquad\qquad\qquad\qquad\qquad{h}^{I}\in {{\mathbb{R}}}^{{\dim }_{{{{{\rm{trans}}}}}.}}\\ I={{{{\rm{Linear}}}}}_{{{{\rm{embed}}}}}({h}^{I})\qquad\qquad\qquad\qquad\qquad\qquad\qquad\qquad\quad I\in {{\mathbb{R}}}^{64}\end{array}$$

We chose this average pooling method primarily because a previous study^21^ found that this resulted in the highest-performing SBERT embeddings. Another alternative would be to simply use the final hidden representation of the ‘cls’ token as a summary of the information in the entire sequence (given that BERT architectures are bidirectional, this token will have access to the whole sequence).

$$\begin{array}{ll}{h}^{\rm{tran.}}={{{\rm{transformer}}}}\Big(\,{{\mbox{[cls]}}}\,,{w}_{1}\ldots {w}_{{{{\mathcal{T}}}}},\,{{\mbox{[eos]}}}\,\Big),\qquad{h}^{\rm{tran.}}\in {{\mathbb{R}}}^{{\dim }_{{{{{\rm{trans}}}}}.}\times {{\,{\mathcal{T}}}}+2}\\ {h}^{I}=({h}_{{{{\rm{cls}}}}}^{\rm{tran.}})\qquad\qquad\qquad\qquad\qquad\qquad\quad\qquad\quad\;\;{h}^{I}\in {{\mathbb{R}}}^{{\dim }_{{{{{\rm{trans}}}}}.}}\end{array}$$

Where ${h}_{{{{\rm{cls}}}}}^{\rm{tran.}}$ denote the last hidden representation for the ‘cls’ token. Ref. ^21^ found this pooling method performed worse than average pooling, so we don’t include these alternatives in our results. For GPT and GPT (XL), we also tested a pooling method where the fixed-length representation for a sequence was taken from the transformer output of the ‘eos’ token. In this case:

$$\begin{array}{ll}{h}^{\rm{tran.}}={{{\rm{transformer}}}}\Big(\,{{\mbox{[cls]}}}\,,{w}_{1}\ldots {w}_{{{{\mathcal{T}}}}},\,{{\mbox{[eos]}}}\,\Big),\qquad{h}^{\rm{tran.}}\in {{\mathbb{R}}}^{{\dim }_{{{{\rm{trans}}}}.}\times {{\;{\mathcal{T}}}}+2}\\ {h}^{I}=({h}_{{{{\rm{eos}}}}}^{\rm{tran.}}),\qquad\qquad\qquad\qquad\qquad\qquad\qquad\quad\quad{h}^{I}\in {{\mathbb{R}}}^{{\dim }_{{{{\rm{trans}}}}.}}\\ I={{{{\rm{Linear}}}}}_{{{{\rm{embed}}}}}({h}^{I}),\qquad\qquad\qquad\qquad\qquad\qquad\quad I\in {{\mathbb{R}}}^{64}\end{array}$$

We found that GPT failed to achieve even a relaxed performance criterion of 85% across tasks using this pooling method, and GPT (XL) performed worse than with average pooling, so we omitted these models from the main results (Supplementary Fig. 11). For CLIP models we use the same pooling method as in the original multiModal training procedure, which takes the outputs of the [cls] token as described above.

For all the above models, we also tested a version where the information from the pretrained transformers is passed through a multilayer perceptron with a single hidden layer of 256 hidden units and ReLU nonlinearities. We found that this manipulation reduced performance across all models, verifying that a simple linear embedding is beneficial to generalization performance.

For GPT, BERT and SBERT, ${\dim }_{{{{\rm{trans}}}}.}=768$ and each model uses a total of ~100 million parameters; for SBERT (L) ${\dim }_{{{{\rm{trans}}}}.}=1,024$ and the model uses ~300 million parameters; GPT (XL) ${\dim }_{{{{\rm{trans}}}}.}=1,600$ and the model uses ~1.5 billion parameters; for CLIP, ${\dim }_{{{{\rm{trans}}}}.}=512$ and the model uses ~60 million parameters. Full PyTorch implementations, including all pretrained weights and model hyperparameters, can be accessed at the Huggingface library (https://huggingface.co/docs/transformers/)^52^.

#### BoW model

For our BoW model, instructions are represented as a vector of binary activations the size of the instruction vocabulary, where each unit indicates the inclusion or exclusion of the associated word in the current instruction. For our instruction set, ∣vocab∣ = 181. This vector is then projected through a linear layer into 64-dimensional space.

$$\begin{array}{ll}{h}_{i}^{{{{\rm{BoW}}}}}=\left\{\begin{array}{ll}1\quad\,{{\mbox{if}}}\,\,{w}_{i}\in ({w}_{1}\ldots {w}_{{{{\mathcal{T}}}}})\\ 0\quad\,{{\mbox{otherwise}}}\,\end{array}\right.\qquad\qquad{h}^{{{{\rm{BoW}}}}}\in {{\mathbb{R}}}^{| \rm{vocab}| }\\ I={{{{\rm{Linear}}}}}_{{{{\rm{embed}}}}}({h}^{{{{\rm{BoW}}}}}),\qquad\qquad\qquad\qquad\qquad\quad I\in {{\mathbb{R}}}^{64}\end{array}$$

#### Blank slate language models

Given that tuning the last layers of language models resulted in improved performance (Fig. 2e), we tested two additional models to determine if training a blank slate language model trained exclusively on the loss from sensorimotor tasks would improve performance. These models consist of passing BoW representations through a multilayer perceptron and passing pretrained BERT word embeddings through one layer of a randomly initialized BERT encoder. Both models performed poorly compared to pretrained models (Supplementary Fig. 4.5), confirming that language pretraining is essential to generalization.

### Tasks sets

Tasks were divided into five interrelated subgroups: ‘go’, ‘decision-making’, ‘matching’, and ‘comparison’ and ‘duration’. Depending on the task, multiple stimuli may appear during the stimulus epoch. Also, depending on the task, models may be required to respond in a particular direction or repress response altogether. Unless otherwise specified, zero-mean Gaussian noise is added independently at each time step and to each input unit and the variance of this noise is drawn randomly from ${\mathbb{U}}[0.1,0.15]$. The timing of stimuli differs among the tasks type. However, for all tasks, trials can be divided into preparatory, stimulus and response epochs. The stimulus epoch can be subdivided into three parts—stim1, delay and stim23—although these distinct parts aren’t used by all tasks. A trial lasts for a total of *T* = 150 time steps. Let *dur*_epoch_ denote the duration in simulated time steps of a given epoch. Then

$$\begin{array}{rcl}&&du{r}_{{{{\rm{response}}}}} \sim\Big\{i| 20 < i\le 25;i\in {\mathbb{N}}\Big\}\\ &&du{r}_{{{{\rm{stim}}}}1},du{r}_{{{{\rm{stim}}}}2} \sim\Big\{i| 37 < i\le 50;i\in {\mathbb{N}}\Big\}\\ &&du{r}_{{{{\rm{delay}}}}} \sim\Big\{i| 15 < i\le 25;i\in {\mathbb{N}}\Big\}\\ &&du{r}_{{{{\rm{prep}}}}.}=150-\Big(du{r}_{{{{\rm{response}}}}}+du{r}_{{{{\rm{stim}}}}1}+du{r}_{{{{\rm{stim}}}}2}+du{r}_{{{{\rm{delay}}}}}\Big)\end{array}$$

For tasks that don’t utilize a delay structure, stim1, stim2 and delay epochs are grouped together in a single stimulus epoch where $du{r}_{{{{\rm{stimulus}}}}}=du{r}_{{{{\rm{stim}}}}1}+du{r}_{{{{\rm{stim}}}}2}+du{r}_{{{{\rm{delay}}}}}$. Unless otherwise specified, a fixation cue with a constant strength *str*_fix_ = 1 is activated throughout the preparatory and stimulus epochs. For example trials of each task, see Supplementary Fig. 13.

#### ‘Go’ tasks

The ‘Go’ family of tasks includes ‘Go’, ‘RTGo’, ‘AntiGo’, ‘AntiRTGo’ and modality-specific versions of each task denoted with either ‘Mod1’ and ‘Mod2’. In both the ‘Go’ and ‘AntiGo’ tasks, a single stimulus is presented at the beginning of the stimulus epoch. The direction of the presented stimulus is generated by drawing from a uniform distribution between 0 and 2*π*, that is, ${\theta }_{{{{\rm{stim}}}}} \sim {\mathbb{U}}[0,2\pi ]$. The stimulus will appear in either modality 1 or modality 2 with equal probability. The strength of the stimulus is given by $st{r}_{{{{\rm{stim}}}}} \sim {\mathbb{U}}[1.0,1.2]$. In the ‘Go’ task, the target response is in the same direction as the presented stimulus, that is, ${\theta }_{{{{\rm{stim}}}}}={\theta }_{{{{\rm{target}}}}}$, while in the ‘AntiGo’ task the direction of the response should be in the opposite of the stimulus direction, ${\theta }_{{{{\rm{stim}}}}}+\pi ={\theta }_{{{{\rm{target}}}}}$. For modality-specific versions of each task, a stimulus direction is drawn in each modality ${\theta }_{{{{\rm{stim}}}},{{{\rm{mod}}}}1} \sim {\mathbb{U}}[0,2\pi ]$ and ${\theta }_{{{{\rm{stim}}}},{{{\rm{mod}}}}2} \sim {\mathbb{U}}[0,2\pi ]$ and for modality-specific Go-type tasks

$${\theta }_{{{{\rm{target}}}}}=\left\{\begin{array}{ll}{\theta }_{{{{\rm{stim}}}},{{{\rm{mod}}}}1} &{{\mbox{if}}}\,\,\,{{\mbox{Mod1 task}}}\\ {\theta }_{{{{\rm{stim}}}},{{{\rm{mod}}}}2} &{{\mbox{if}}}\,\,\,{{\mbox{Mod2 task}}}\end{array}\right.$$

while for modality-specific AntiGo-type tasks

$${\theta }_{{{{\rm{target}}}}}=\left\{\begin{array}{ll}{\theta }_{{{{\rm{stim}}}},{{{\rm{mod}}}}1}+\pi &{{\mbox{if}}}\,\,\,{{\mbox{Mod1 task}}}\,\\ {\theta }_{{{{\rm{stim}}}},{{{\rm{mod}}}}2}+\pi &{{\mbox{if}}}\,\,\,{{\mbox{Mod2 task}}}\end{array}\right.$$

For ‘RT’ versions of the ‘Go’ tasks, stimuli are only presented during the response epoch and the fixation cue is never extinguished. Thus, the presence of the stimulus itself serves as the response cue and the model must respond as quickly as possible. Otherwise, stimuli persist through the duration of the stimulus epoch.

#### ‘Decision-making’ tasks

The ‘decision-making’ family of tasks includes ‘DM’ (decision-making), ‘AntiDM’, ‘MultiDM’ (multisensory decision-making), ‘AntiMultiDM,’ modality-specific versions of each of these tasks and, finally, confidence-based versions of ‘DM’ and ‘AntiDM.’ For all tasks in this group, two stimuli are presented simultaneously and persist throughout the duration of the stimulus epoch. They are drawn according to $${\theta }_{{{{\rm{stim}}}}1} \sim {\mathbb{U}}[0,2\pi ]$$ and $${\theta }_{{{{\rm{stim}}}}2} \sim {\mathbb{U}}$$$$[({\theta }_{{{{\rm{stim}}}}1}-0.2\pi ,{\theta }_{{{{\rm{stim}}}}1}-0.6\pi )\cup ({\theta }_{{{{\rm{stim}}}}1}+0.2\pi ,{\theta }_{{{{\rm{stim}}}}1}+0.6\pi )]$$. A base strength applied to both stimuli is drawn such that $$st{r}_{\rm{base}} \sim {\mathbb{U}}[1.0,1.2]$$. A contrast is drawn from a discrete distribution such that *c* ~ {−0.175, −0.15, −0.1, 0.1, 0.15, 0.175} so the stimulus strength associated with each direction in a trial are given by $$st{r}_{{{{\rm{stim}}}}1}=st{r}_{\rm{base}}+c$$ and $$st{r}_{{{{\rm{stim}}}}2}=$$ $${str}_{\rm{base}}-c$$.

For the ‘DM’ task,

$${\theta }_{{{{\rm{target}}}}}=\left\{\begin{array}{ll}{\theta }_{{{{\rm{stim}}}}1}\quad &\,{{\mbox{if}}}\,\,st{r}_{{{{\rm{stim}}}}1} > st{r}_{{{{\rm{stim}}}}2}\\ {\theta }_{{{{\rm{stim}}}}2}\quad &\,{{\mbox{otherwise}}}\,\end{array}\right.$$

and for the the ‘AntiDM’ task,

$${\theta }_{{{{\rm{target}}}}}=\left\{\begin{array}{ll}{\theta }_{{{{\rm{stim}}}}1}\quad &\,{{\mbox{if}}}\,\,st{r}_{{{{\rm{stim}}}}1} < st{r}_{{{{\rm{stim}}}}2}\\ {\theta }_{{{{\rm{stim}}}}2}\quad &\,{{\mbox{otherwise}}}\,\end{array}\right.$$

For these versions of the tasks, the stimuli are presented in either modality 1 or modality 2 with equal probability. For the multisensory versions of each task, stimuli directions are drawn in the same manner and presented across both modalities so that $${\theta }_{{{{\rm{stim}}}}1,{{{\rm{mod}}}}1}={\theta }_{{{{\rm{stim}}}}1,{{{\rm{mod}}}}2}$$ and $${\theta }_{{{{\rm{stim}}}}2,{{{\rm{mod}}}}1}={\theta }_{{{{\rm{stim}}}}2,{{{\rm{mod}}}}2}$$. Base strengths are drawn independently for each modality. Contrasts for both modalities are drawn from a discrete distribution such that $${c}_{{{\mathrm{mod}}}\,1},{c}_{{{\mathrm{mod}}}\,2} \sim \left\{0.2,0.175,\right.$$$$\left.0.15,0.125,-0.125,-0.15,-0.175,-0.2\right\}$$. If both $$| {c}_{{{\mathrm{mod}}}\,1}| -| {c}_{{{\mathrm{mod}}}\,2}| =0$$ then contrasts are redrawn to avoid zero-contrast trials during training. If both $${c}_{{{\mathrm{mod}}}\,1}$$ and $${c}_{{{\mathrm{mod}}}\,2}$$ have the same sign, then contrasts are redrawn to ensure that the trial requires integrating over both modalities as opposed to simply performing a ‘DM’ task in a single modality. Criteria for target responses are measured as the strength of a given direction summed over both modalities. So, for ‘MultiDM’

$${\theta }_{{{{\rm{target}}}}}=\left\{\begin{array}{ll}{\theta }_{{{{\rm{stim}}}}1,{{\mathrm{mod}}}\,1}\quad &\,{{\mbox{if}}}\,\,st{r}_{{{{\rm{stim}}}}1,{{{\rm{mod}}}}1}+st{r}_{{{{\rm{stim}}}}1,{{{\rm{mod}}}}2} > st{r}_{{{{\rm{stim}}}}2,{{{\rm{mod}}}}1}\\&+st{r}_{{{{\rm{stim}}}}2,{{{\rm{mod}}}}2}\\ {\theta }_{{{{\rm{stim}}}}2,{{{\rm{mod}}}}1}\quad &\,{{\mbox{otherwise}}}\,\end{array}\right.$$

and for ‘AntiMultiDM’

$${\theta }_{{{{\rm{target}}}}}=\left\{\begin{array}{ll}{\theta }_{{{{\rm{stim}}}}1,{{\mathrm{mod}}}\,1}\quad &\,{{\mbox{if}}}\,\,st{r}_{{{{\rm{stim}}}}1,{{{\rm{mod}}}}1}+st{r}_{{{{\rm{stim}}}}1,{{{\rm{mod}}}}2} < st{r}_{{{{\rm{stim}}}}2,{{{\rm{mod}}}}1}\\&+st{r}_{{{{\rm{stim}}}}2,{{{\rm{mod}}}}2}\\ {\theta }_{{{{\rm{stim}}}}2,{{{\rm{mod}}}}1}\quad &\,{{\mbox{otherwise}}}\,\end{array}\right.$$

Stimuli for modality-specific versions of each task are generated in the same way as multisensory versions of the task. Criteria for target response are the same as standard versions of ‘DM’ and ‘AntiDM’ tasks applied only to stimuli in the relevant modality.

In confidence-based decision-making tasks (‘ConDM’ and ‘ConAntiDM’), the stimuli directions are drawn in the same way as above. Stimuli are shown in either modality 1 or modality 2 with equal probability. In each trial, *str*_base_ = 1. The contrast and noise for each trial is based on the thresholded performance of a SIMPLENET model trained on all tasks except ‘ConDM’ and ‘ConAntiDM’. Once this model has been trained, we establish a threshold across levels of noise and contrasts for which the model can perform a ‘DM’ or an ‘AntiDM’ task at 95% correct. We then draw contrasts and noises for trials from above and below this threshold with equal probability during training. In trials where the noise and contrast levels fell below the 95% correct threshold, the model must repress response, and otherwise perform the decision-making task (either ‘DM’ or ‘AntiDM’).

#### ‘Comparison’ tasks

Our comparison task group includes ‘COMP1’, ‘COMP2’, ‘MultiCOMP1’, ‘MultiCOMP2’, ‘Anti’ versions of each of these tasks, as well as modality-specific versions of ‘COMP1’ and ‘COMP2’ tasks. This group of tasks is designed to extend the basic decision-making framework into a setting with more complex control demands. These tasks utilize the delay structure in the stimulus epoch so that stim1 appears only during the stim1 epoch, followed by a delay, and finally stim2. This provides a temporal ordering on the stimuli. In ‘COMP1’, the model must respond to the first stimulus only if it has greater strength than the second and otherwise repress a response that is

$${\theta }_{{{{\rm{target}}}}}=\left\{\begin{array}{ll}{\theta }_{{{{\rm{stim}}}}1}\quad &\,{{\mbox{if}}}\,\,st{r}_{{{{\rm{stim}}}}1} > st{r}_{{{{\rm{stim}}}}2}\\ {{{\rm{repress}}}}\quad &\,{{\mbox{otherwise}}}\,\end{array}\right.$$

Likewise, in ‘COMP2’, the model must respond to the second direction if it presented with greater strength than the first otherwise repress response that is

$${\theta }_{{{{\rm{target}}}}}=\left\{\begin{array}{ll}{\theta }_{{{{\rm{stim}}}}2}\quad &\,{{\mbox{if}}}\,\,st{r}_{{{{\rm{stim}}}}2} > {{{{\rm{str}}}}}_{{{{\rm{stim}}}}1}\\ {{{\rm{repress}}}}\quad &\,{{\mbox{otherwise}}}\,\end{array}\right.$$

In ‘Anti’ versions of the task the ordering criteria is the same except for stimuli with least strength, that is, for ‘AntiCOMP1’

$${\theta }_{{{{\rm{target}}}}}=\left\{\begin{array}{ll}{\theta }_{{{{\rm{stim}}}}1}\quad &\,{{\mbox{if}}}\,\,{{{{\rm{str}}}}}_{{{{\rm{stim}}}}1} < {{{{\rm{str}}}}}_{{{{\rm{stim}}}}2}\\ {{{\rm{repress}}}}\quad &\,{{\mbox{otherwise}}}\,\end{array}\right.$$

and for ‘AntiCOMP2’

$${\theta }_{{{{\rm{target}}}}}=\left\{\begin{array}{ll}{\theta }_{{{{\rm{stim}}}}2}\quad &\,{{\mbox{if}}}\,\,{{{{\rm{str}}}}}_{{{{\rm{stim}}}}2} < {{{{\rm{str}}}}}_{{{{\rm{stim}}}}1}\\ {{{\rm{repress}}}}\quad &\,{{\mbox{otherwise}}}\,\end{array}\right.$$

In multisensory settings, the criteria for target direction are analogous to the multisensory decision-making tasks where strength is integrated across modalities. Likewise, for modality-specific versions, the criteria are only applied to stimuli in the relevant modality. Stimuli directions and strength for each of these tasks are drawn from the same distributions as the analogous task in the ‘decision-making’ family. However, during training, we make sure to balance trials where responses are required and trials where models must repress response.

#### ‘Duration’ tasks

The ‘duration’ family of tasks includes ‘Dur1’, ‘Dur2’, ‘MultiDur1’, ‘MultiDur2’, ‘Anti’ versions of each of these tasks and modality-specific versions of ‘Dur1’ and ‘Dur2’ tasks. These tasks require models to perform a time estimation task with the added demand or stimuli ordering determining relevance for response. Like in ‘comparison’ tasks, stim1 is presented followed by a delay and then stim2. For ‘Dur1’ trials

$${\theta }_{{{{\rm{target}}}}}=\left\{\begin{array}{ll}{\theta }_{{{{\rm{stim}}}}1}\quad &\,{{\mbox{if}}}\,\,du{r}_{{{{\rm{stim}}}}1} > du{r}_{{{{\rm{stim}}}}2}\\ {{{\rm{repress}}}}\quad &\,{{\mbox{otherwise}}}\,\end{array}\right.$$

Likewise, for ‘Dur2’

$${\theta }_{{{{\rm{target}}}}}=\left\{\begin{array}{ll}{\theta }_{{{{\rm{stim}}}}2}\quad &\,{{\mbox{if}}}\,\,du{r}_{{{{\rm{stim}}}}2} > du{r}_{{{{\rm{stim}}}}1}\\ {{{\rm{repress}}}}\quad &\,{{\mbox{otherwise}}}\,\end{array}\right.$$

In ‘Anti’ versions of these tasks, the correct response is in the direction of the stimulus with the shortest duration given the ordering criteria is met. Hence, for ‘AntiDur1’

$${\theta }_{{{{\rm{target}}}}}=\left\{\begin{array}{ll}{\theta }_{{{{\rm{stim}}}}1}\quad &\,{{\mbox{if}}}\,\,du{r}_{{{{\rm{stim}}}}1} < du{r}_{{{{\rm{stim}}}}2}\\ {{{\rm{repress}}}}\quad &\,{{\mbox{otherwise}}}\,\end{array}\right.$$

and for ‘AntiDur2’

$${\theta }_{{{{\rm{target}}}}}=\left\{\begin{array}{ll}{\theta }_{{{{\rm{stim}}}}2}\quad &\,{{\mbox{if}}}\,\,du{r}_{{{{\rm{stim}}}}2} < du{r}_{{{{\rm{stim}}}}1}\\ {{{\rm{repress}}}}\quad &\,{{\mbox{otherwise}}}\,\end{array}\right.$$

Across these tasks directions are drawn according to ${\theta }_{{{{\rm{stim}}}}1} \sim {\mathbb{U}}[0,2\pi ]$ and ${\theta }_{{{{\rm{stim}}}}2} \sim {\mathbb{U}}[({\theta }_{{{{\rm{stim}}}}1}-0.2\pi ,{\theta }_{{{{\rm{stim}}}}1}-0.6\pi )\cup ({\theta }_{{{{\rm{stim}}}}1}+0.2\pi ,{\theta }_{{{{\rm{stim}}}}1}+0.6\pi )]$. Stimulus strengths are drawn according to $st{r}_{{{{\rm{stim}}}}1},st{r}_{{{{\rm{stim}}}}2} \sim {\mathbb{U}}[0.8,1.2]$. To set the duration of each stimulus, we first draw $du{r}_{{{{\rm{long}}}}} \sim$ $\{i| 35 < i\le 50,i\in {\mathbb{N}}\}$ and $du{r}_{{{{\rm{short}}}}} \sim \{i| 25 < i\le (du{r}_{{{{\rm{long}}}}}-8),i\in {\mathbb{N}}\}$. During training, we determine which trials for a given task should and should not require a response in order to evenly balance repress and respond trials. We then assign *dur*_long_ and *dur*_short_ to either stim1 or stim2 so that the trial requires the appropriate response given the particular task type.

Again, criteria for correct response in the multisensory and modality-specific versions of each tasks follow analogous tasks in the ‘decision-making’ and ‘comparison’ groups where multisensory versions of the task require integrating total duration over each modality, and modality-specific tasks require only considering durations in the given task modality. For multisensory tasks, we draw duration value $du{r}_{{{{\rm{long}}}}} \sim \{i| 75 < i\le 100,i\in {\mathbb{N}}\}$ and then split this value *dur*_long0_ = *dur*_long _× 0.55 and *dur*_long1_ = *dur*_long _× 0.45. We also draw a value *dur*_short_ = *dur*_long_ − Δ*dur* where $\Delta dur \sim \{i| 15 < i\le 25,i\in {\mathbb{N}}\}$. This value is then subdivided further into *dur*_short0_ = *dur*_long1_ + Δ*dur*_short_ where $\Delta du{r}_{{{{\rm{short}}}}} \sim$ $\{i| 19 < i\le 15,i\in {\mathbb{N}}\}$ and *dur*_short1_ = *dur*_Short_ − *dur*_short0_. Short and long durations can then be allocated to the ordered stimuli according to task type. Drawing durations in this manner ensures that, like in ‘decision-making’ and ‘comparison’ groups, correct answers truly require models to integrate durations over both modalities, rather than simply performing the task in a given modality to achieve correct responses.

#### ‘Matching’ tasks

The ‘matching’ family of tasks consists of ‘DMS’ (delay match to stimulus), ‘DNMS’ (delay non-match to stimulus), ‘DMC’ (delay match to category) and ‘DMNC’ (delay non-match to category) tasks. For all tasks, stim1 is presented at the beginning of the stimulus epoch, followed by a delay, and the presentation of stim2. The stimulus strength is drawn according to $st{r}_{{{{\rm{stim}}}}1},st{r}_{{{{\rm{stim}}}}2} \sim {\mathbb{U}}[0.8,1.2]$. The input modality for any given trial is chosen at random with equal probability. In both ‘DMS’ and ‘DNMS’ tasks, trials are constructed as ‘matching stim’ trials or ‘mismatching stim’ trials with equal probability. In ‘matching stim’ trials ${\theta }_{{{{\rm{stim}}}}1} \sim {\mathbb{U}}[0,2\pi ]$ and ${\theta }_{{{{\rm{stim}}}}2}={\theta }_{{{{\rm{stim}}}}1}$. In ‘mismatch stim’ trials, ${\theta }_{{{{\rm{stim}}}}1} \sim {\mathbb{U}}[0,2\pi ]$ and

$${\theta }_{{{{\rm{stim}}}}2} \sim {\mathbb{U}}[({\theta }_{{{{\rm{stim}}}}1}-0.2\pi ,{\theta }_{{{{\rm{stim}}}}1}-0.6\pi )\cup ({\theta }_{{{{\rm{stim}}}}1}+0.2\pi ,{\theta }_{{{{\rm{stim}}}}1}+0.6\pi )].$$

For ‘DMS’, models must respond in the displayed direction if the stimuli match, otherwise repress response,

$${\theta }_{{{{\rm{target}}}}}=\left\{\begin{array}{ll}{\theta }_{{{{\rm{stim}}}}1}\quad &\,{{\mbox{if}}}\,\,{\theta }_{{{{\rm{stim}}}}1}={\theta }_{{{{\rm{stim}}}}2}\\ {{{\rm{repress}}}}\quad &\,{{\mbox{otherwise}}}\,\end{array}\right.$$

and for ‘DNMS’, models must respond to the second direction if both directions are mismatched,

$${\theta }_{{{{\rm{target}}}}}=\left\{\begin{array}{ll}{\theta }_{{{{\rm{stim}}}}2}\quad &\,{{\mbox{if}}}\,\,{\theta }_{{{{\rm{stim}}}}1}\ne {\theta }_{{{{\rm{stim}}}}2}\\ {{{\rm{repress}}}}\quad &\,{{\mbox{otherwise}}}\,\end{array}\right.$$

‘DMC’ and ‘DNMC’ tasks are organized in a similar manner. The stimulus input space is divided evenly into two categories such that cat1 = {*θ*: 0 < *θ*≤*π*} and cat2 = {*θ*: *π* < *θ*≤2*π*}. For ‘DMC’ and ‘DNMC’ tasks, trials are constructed as ‘matching cat.’ trials or ‘mismatching cat.’ trials with equal probability. In ‘matching cat.’ trials ${\theta }_{{{{\rm{stim}}}}1} \sim {\mathbb{U}}[0,2\pi ]$ and ${\theta }_{{{{\rm{stim}}}}2} \sim {\mathbb{U}}({{{\mbox{cat}}}}_{{{{\rm{stim}}}}1})$, where ${\mathbb{U}}({{{\mbox{cat}}}}_{{{{\rm{stim}}}}1})$ is a uniform draw from the category of stim1. In ‘mismatch stim’ trials, ${\theta }_{{{{\rm{stim}}}}1} \sim {\mathbb{U}}[0,2\pi ]$ and ${\theta }_{{{{\rm{stim}}}}2} \sim {\mathbb{U}}(-{{{\mbox{cat}}}}_{{{{\rm{stim}}}}1})$ where $-{{{\mbox{cat}}}}_{{{{\rm{stim}}}}1}$ is the opposite category as stim1. For ‘DMC’, the model must respond in the first direction if both stimuli are presented in the same category otherwise repress response,

$${\theta }_{{{{\rm{target}}}}}=\left\{\begin{array}{ll}{\theta }_{{{{\rm{stim}}}}1}\quad &\,{{\mbox{if}}}\,\,{{{\mbox{cat}}}}_{{{{\rm{stim}}}}1}={{{\mbox{cat}}}}_{{{{\rm{stim}}}}2}\\ {{{\rm{repress}}}}\quad &\,{{\mbox{otherwise}}}\,\end{array}\right.$$

and for ‘DNMC’, the model should respond to the second direction if both stimuli are presented in opposite categories otherwise repress response,

$${\theta }_{{{{\rm{target}}}}}=\left\{\begin{array}{ll}{\theta }_{{{{\rm{stim}}}}2}\quad &\,{{\mbox{if}}}\,\,{{{\mbox{cat}}}}_{{{{\rm{stim}}}}1}\ne {{{\mbox{cat}}}}_{{{{\rm{stim}}}}2}\\ {{{\rm{repress}}}}\quad &\,{{\mbox{otherwise}}}\,\end{array}\right.$$

### Target output and correct criteria

The target output $y\in {{\mathbb{R}}}^{33\times T}$ for a trial entails maintaining fixation in *y*_1_ = *y*_fix_ during the stimulus epoch, and then either responding in the correct direction or repressing activity in the remaining target response units *y*_2…33_ in the response epoch. Since the model should maintain fixation until response, target for fixation is set at *y*_fix_ = 0.85 during preparatory and stimulus epochs and *y*_fix_ = 0.05 in the response epoch. When a response is not required, as in the preparatory and stimulus epochs and with repressed activity in the response epoch, unit *i* takes on a target activity of *y*_*i*_ = 0.05. Alternatively, when there is a target direction for response,

$${y}_{i}=0.8\exp \left[-0.5 \times {\left(\frac{8| {\theta }_{{{{\rm{target}}}}}-{\theta }_{i}| }{\pi }\right)}^{2}\right]+0.05$$

where *θ*_*i*_ is the preferred direction for unit *i*. Like in sensory stimuli, preferred directions for target units are evenly spaced values from [0, 2*π*] allocated to the 32 response units.

For a model response to count as correct, it must maintain fixation, that is, ${\hat{y}}_{{{{\rm{fix}}}}} > 0.5$ during preparatory and stimulus epochs. When no response is required ${\hat{y}}_{i} < 0.15$. When a response is required, response activity is decoded using a population vector method and ${\theta }_{{{{\rm{resp}}}}.}\in ({\theta }_{{{{\rm{target}}}}}-\frac{\pi }{10},{\theta }_{{{{\rm{target}}}}}+\frac{\pi }{10})$. If the model fails to meet any of these criteria, the trial response is incorrect.

### Model training

Again following ref. ^18^, model parameters are updated in a supervised fashion according to a masked mean squared error loss (mMSE) computed between the model motor response, ${\hat{y}}_{1\ldots T}=\hat{y}$, and the target, *y*_1…*T*_ = *y*, for each trial.

$$L={{{\rm{mMSE}}}}(\,y,\hat{y})={\rm{mask}} \times {\Big\langle {\left({\,y}_{t}-{{\hat{y}_{t}}}\right)}^{2}\Big\rangle }_{t}$$

Here, the multiplication sign denotes element-wise multiplication. Masks weigh the importance of different trial epochs. During preparatory and stimulus epochs, mask weights are set to 1; during the first five time steps of the response epoch, the mask value is set to 0; and during the remainder of the response epoch, the mask weight is set to 5. The mask value for the fixation is twice that of other values at all time steps.

For all models, we update Θ = {sensorimotor-RNN, Linear_out_} during training on our task set. For instructed models, we additionally update Linear_embed_ in the process of normal training. We train models using standard PyTorch machinery and an Adam optimizer. An epoch consists of 2,400 mini-batches, with each mini-batch consisting of 64 trials. For all models, we use the same initial learning rate as in ref. ^18^, *lr* = 0.001. We found that in the later phases of training, model performance oscillated based on which latest task presented during training, so we decayed the learning rate for each epoch by a factor of *γ* = 0.95, which allowed performance to converge smoothly. Following ref. ^18^, models train until they reach a threshold performance of 95% across all tasks (and train for a minimum of 35 epochs). We found that training for GPTNET tended to asymptote below performance threshold for multisensory versions of comparison tasks. This held true over a variety of training hyperparameters and learning rate scheduler regimes. Hence, we relax the performance threshold of GPTNET to 85%. For each model type, we train five models that start from five different random initializations. Where applicable, results are averaged over these initializations.

#### Language model fine-tuning

When fine-tuning models, we allow the gradient from the motor loss experienced during sensorimotor training to fine-tune the weights in the final layers of the transformer language models. During normal training, we checkpoint a copy of our instructed models after training for 30 epochs. We then add the last three transformer layers to the set of trainable parameters, and reset the learning rates to *lr* = 1 × 10^−^^4^ for Θ = {sensorimotor-RNN, Linear_out_} and *lr*^lang^ = 3 × 10^−4^ for Θ^lang^ = {Linear_embed_, transformer_−3,−2,−1_} where transformer_−3,−2,−1_ denotes the parameters of the last three layers of the relevant transformer architecture. We used these reduced learning rates to avoid completely erasing preexisting linguistic knowledge. Similarly for RNN parameters, we found the above learning rate avoided catastrophic forgetting of sensorimotor knowledge while also allowing the RNN to adapt to updated language embeddings across all models. Autoregressive models were much more sensitive to this procedure, often collapsing at the beginning of fine-tuning. Hence, for GPTNETXL and GPTNET, we used *lr*^lang^ = 5 × 10^−5^, which resulted in robust learning. Models train until they reach a threshold performance of 95% across training tasks or 85% correct for GPTNET.

### Hold-out testing

During hold-out testing, we present models with 100 batches of one of the tasks that had been held out of training. For the instructed model, the only weights allowed to update during this phase are Θ = {sensorimotor-RNN, Linear_out_, Linear_embed_}. All weights of SIMPLENET and STRUCTURENET are trainable in this context. In this hold-out setting, we found that in more difficult tasks for some of our more poorly performing models, the standard hyperparameters we used during training resulted in unstable learning curves for novel tasks. To stabilize performance and thereby create fair comparisons across models, we used an increased batch size of 256. We then began with the standard learning rate of 0.001 and decreased this by increments of 0.0005 until all models showed robust learning curves. This resulted in a learning rate of 8 × 10^−4^. All additional results shown in the Supplementary Information section 4 follow this procedure.

### CCGP calculation

To calculate CCGP, we trained a linear decoder on a pair of tasks and then tested that decoder on alternative pairs of tasks that have an analogous relationship. We grouped tasks into eight dichotomies: ‘Go’ versus ‘Anti’, ‘Standard’ versus ‘RT’, ‘Weakest’ versus ‘Strongest’, ‘Longest’ versus ‘Shortest’, ‘First Stim.’ versus ‘Second Stim’, ‘Stim Match’ versus ‘Category Match’, ‘Matching’ versus ‘Non-Matching’ and ‘Mod1’ versus ‘Mod2’. As an example, the ‘Go’ versus ‘Anti’ dichotomy includes (‘Go’, ‘AntiGo’), (‘GoMod1’, ‘AntiGoMod1’), (‘GoMod2’, ‘AntiGoMod2’), (‘RTGo’, ‘AntiRTGo’), (‘RTGoMod1’, ‘AntiRTGoMod1’) and (‘RTGoMod2’, ‘AntiRTGoMod2’) task pairs. For ‘RNN’ task representations, we extracted activity at the time of stimulus onset for 250 example trials. For language representations, we input the instruction sets for relevant tasks to our language model and directly analyze activity in the ‘embedding’ layer or take the sequence-averaged activity in each transformer layer. For nonlinguistic models, we simply analyze the space of rule vectors. Train and test conditions for decoders were determined by dichotomies identified across the task set (Supplementary Note 1). To train and test decoders, we used sklearn.svm.LinearSVC Python package. The CCGP score for a given task is the average decoding score achieved across all dichotomies where the task in question was part of either the train set or the test set. For model scores reported in the main text, we only calculate CCGP scores for models where the task in question has been held out of training. In Supplementary Fig. 9, we report scores on tasks where models have been trained on all tasks, and for models where instructions have been switched for the hold-out task.

For Fig. 3e, we calculated Pearson’s *r* correlation coefficient between performance on held-out tasks and CCGP scores per task, as well as a *P*-value testing against the null hypothesis that these metrics are uncorrelated and normally distributed (using the scipy.stats.pearsonr function). Full statistical tests for CCGP scores of both RNN and embedding layers from Fig. 3f can be found in Supplementary Fig. 9. Note that transformer language models use the same set of pretrained weights among random initialization of Sensorimotor-RNNs, thus for language model layers, the Fig. 3f plots show the absolute scores of those language models.

### Conditional clause/deduction task analysis

We first split our task set into two groups (listed below): tasks that included conditional clauses and simple deductive reasoning components (30 tasks) and those where instructions include simple imperatives (20 tasks). We computed the difference in performance across the mean of generalization performance for each group across random initialization for each model (Fig. 2f). We compared these differences to a null distribution constructed by performing a set of 50 random shuffles of the task set into groups of 30 and 20 tasks and computing differences in the same way, again using two-sided unequal-variance *t*-tests. Because STRUCUTRENET is a nonlinguistic model, we then compared performance of STRUCUTRENET to our instructed models to disassociate the effects of performing tasks with a deductive reasoning component versus processing instructions with more complicated conditional clause structure. Results of all statistical tests are reported in Supplementary Fig. 6).

Simple imperative tasks include: ‘Go’, ‘AntiGo’, ‘RTGo’, ‘AntiRTGo’, ‘GoMod1’, ‘GoMod2’, ‘AntiGoMod1’, ‘AntiGoMod2’, ‘RTGoMod1’, ‘AntiRTGoMod2’, ‘RTGoMod2’, ‘AntiRTGoMod2’, ‘DM’, ‘AntiDM’, ‘MultiDM’, ‘AntiMultiDM’, ‘DMMod1’, ‘DMMod2’, ‘AntiDMMod1’ and ‘AntiDMMod2’.

Conditional clause/deduction tasks include: ‘ConDM’, ‘ConAntiDM’, ‘Dur1’, ‘Dur2’, ‘MultiDur1’, ‘MultiDur2’, ‘AntiDur1’, ‘AntiDur2’, ‘AntiMultiDur1’, ‘AntiMultiDur2’, ‘Dur1Mod1’, ‘Dur1Mod2’, ‘Dur2Mod1’, ‘Dur2Mod2’, ‘COMP1’, ‘COMP2’, ‘MultiCOMP1’, ‘MultiCOMP2’, ‘AntiCOMP1’, ‘AntiCOMP2’, ‘AntiMultiCOMP1’, ‘AntiMultiCOMP2’, ‘COMP1Mod1’, ‘COMP1Mod2’, ‘COMP2Mod1’, ‘COMP2Mod2’, ‘DMS’, ‘DNMS’, ‘DMC’ and ‘DMNC’.

### Language production training

#### Self-supervised language production network training

Our language production framework is inspired by classic sequence-to-sequence modeling using RNNs^53^. Our Production-RNN is a GRU with 256 hidden units using ReLU nonlinearities. At each step in the sequence, a set of decoder weights, Linear_words_, attempts to decode the next token, *w*_*τ*+1_, from the hidden state of the recurrent units. The hidden state of the Production-RNN is initialized by concatenating the time average and maximum sensorimotor activity of a SBERTNET (L) and passing that through weights Linear_sm_. The linguistic instruction used to drive the initializing sensorimotor activity is in turn used as the target set of tokens for the Production-RNN outputs. The first input to the Production-RNN is always a special start-of-sentence token, and the decoder runs until an end-of-sentence token is decoded or until input reaches a length of 30 tokens. Suppose ${w}_{1,k}\ldots {w}_{{{{\mathcal{T}}}},k}\in {\rm{Instruc{t}}}_{k}^{i}$ is the sequence of tokens in instruction *k* where *k* is in the instruction set for task *i* and *X*^*i*^ is sensory input for a trial of task *i*. For brevity, we denote the process by which language models embed instructions as Embed() (see ‘Pretrained transformers’). The decoded token at the *τ*^th^ position, ${\hat{w}}_{\tau ,k}$, is then given by

$$\begin{array}{ll}{h}_{T}^{sm}={{{\rm{SensorimotorRNN}}}}\left({X}^{i},Embed\left({w}_{1,k}\ldots {w}_{{{{\mathcal{T}}}},k}\right)\right)\quad\quad{h}_{T}^{sm}\in {{\mathbb{R}}}^{T\times 256}\\ sm\_out=\left.\right({{{{\rm{mean}}}}}_{T}\left({h}_{T}^{sm}\right),\mathop{\max }\limits_{T}\left({h}_{T}^{sm}\right)\quad\quad\quad\quad\quad\quad\quad\quad\quad\;\;{sm}\_{out}\in {{\mathbb{R}}}^{512}\\ \overline{{h}_{0}^{{{{\rm{decoder}}}}}}={{{\rm{relu}}}}\left({{{{\rm{Linear}}}}}_{{{{\rm{sm}}}}}(sm\_out)\right)\quad\quad\quad\quad\quad\quad\quad\quad\quad\quad\;\overline{{h}_{0}^{{{{\rm{decoder}}}}}}\in {{\mathbb{R}}}^{256}\\ {h}_{0}^{{{{\rm{decoder}}}}}={{{\rm{Dropout}}}}\left(\overline{{h}_{0}^{{{{\rm{decoder}}}}}}\right)\;\;\;\quad\quad\quad\quad\quad\quad\quad\quad\quad\qquad\quad{h}_{0}^{{{{\rm{decoder}}}}}\in {{\mathbb{R}}}^{256}\\ {h}_{\tau }^{{{{\rm{decoder}}}}}={{{\rm{ProductionRNN}}}}\left({\hat{w}}_{1,k}\ldots {\hat{w}}_{\tau -1,k};{h}_{0}^{{{{\rm{decoder}}}}}\right),\quad\quad\quad{h}_{\tau }^{{{{\rm{decoder}}}}}\in {{\mathbb{R}}}^{256}\\ {p}_{{\hat{w}}_{\tau ,k}}={{{\rm{softmax}}}}\left({{{{\rm{Linear}}}}}_{{{{\rm{words}}}}}\left({h}_{\tau ,k}^{{{{\rm{decoder}}}}}\right)\right)\quad\quad\quad\quad\quad\quad\quad\quad\quad{p}_{{\hat{w}}_{\tau ,k}}\in {{\mathbb{R}}}^{| vocab| },\\ {\hat{w}}_{\tau ,k}={{{\rm{argmax}}}}\left({p}_{{\hat{w}}_{\tau ,k}}\right)\end{array}$$

The model parameters Θ^production^ = {Linear_sm_, Linear_words_, Production-RNN} are trained using cross-entropy loss between the ${p}_{{\hat{w}}_{\tau ,i}}$ and the instruction token *w*_*τ*,*k*_ provided to the sensorimotor-RNN as input. We train for 80 epochs of 2,400 batches with 64 trials per batch and with task type randomly interleaved. We found that using an initial learning rate of 0.001 sometimes caused models to diverge in early phases of training, so we opted for a learning rate of 1× 10^−4^, which led to stable early training. To alleviate similar oscillation problems detected in sensorimotor training, we also decayed the learning rate by *γ* = 0.99 per epoch. Additionally, the use of a dropout layer with a dropout rate of 0.05 improved performance. We also used a teacher forcing curriculum, where for some ratio of training batches, we input the ground truth instruction token *w*_*τ*,*k*_ at each time step instead of the models decoded word ${\hat{w}}_{\tau ,k}$. At each epoch, ${\rm{teacher}}\,{{\mbox{\_}}}{\rm{forcing}}{{\mbox{\_}}}$ ${\rm{ratio}}=0.5 \times \frac{80-{{{\rm{epoch}}}}}{80}$.

#### Obtaining embedding layer activity using motor feedback

For a task, *i*, we seek to optimize a set of embedding activity vectors ${E}^{i}\in {{\mathbb{R}}}^{64}$ such that when they are input as task-identifying information, the model will perform the task in question. Crucially, we freeze all model weights Θ = {sensorimotor-RNN, Linear_out_, Linear_embedding_} and only update *E*^*i*^ according to the standard supervised loss on the motor output. For notional clarity, GRU dependence on the previous hidden state *h*_*t*−1_ has been made implicit in the following equations.

$$\begin{array}{rcl}{\hat{y}}^{i}&=&\sigma \Big({{{{\rm{Linear}}}}}_{{{{\rm{out}}}}}\left({{{\rm{SensorimotorRNN}}}}({X}^{i},{E}^{i})\right)\Big)\\ L&=&{\rm{mMSE}}(y,\hat{y})\end{array}$$

We optimized a set of 25 embedding vectors for each task, again using an Adam optimizer. Here the optimization space has many suboptimal local minimums corresponding to embeddings for related tasks. Hence, we used a high initial learning rate of *lr* = 0.05, which we decayed by *γ* = 0.8 for each epoch. This resulted in more robust learning than lower learning rates. An epoch lasts for 800 batches with a batch length of 64, and we train for a minimum of 1 epoch or until we reach a threshold performance of 90% or 85% on ‘DMC’ and ‘DNMC’ tasks.

#### Producing task instructions

To produce task instructions, we simply use the set *E*^*i*^ as task-identifying information in the input of the sensorimotor-RNN and use the Production-RNN to output instructions based on the sensorimotor activity driven by *E*^*i*^. For each task, we use the set of embedding vectors to produce 50 instructions per task. We repeat this process for each of the 5 initializations of sensorimotor-RNN, resulting in 5 distinct language production networks, and 5 distinct sets of learned embedding vectors. Reported results for each task are averaged over these 5 networks. For the confusion matrix (Fig. 5d), we report the average percentage that decoded instructions are in the training instruction set for a given task or a novel instruction. Partner model performance (Fig. 5e) for each network initialization is computed by testing each of the 4 possible partner networks and averaging over these results.

### Sample sizes/randomization

No statistical methods were used to predetermine sample sizes but following ref. ^18^ we used five different random weight initializations per language model tested. Randomization of weights was carried out automatically in Python and PyTorch software packages. Given this automated randomization of weights, we did not use any blinding procedures in our study. No data were excluded from analyses.

### Software

All simulation and data analysis was performed in Python 3.7.11. PyTorch 1.10 was used to implement and train models (this includes Adam optimizer implementation). Transformers 4.16.2 was used to implement language models and all pretrained weights for language models were taken from the Huggingface repository (https://huggingface.co/docs/transformers/). We also used scikit-learn 0.24.1 and scipy 1.7.3 to perform analyses.

### Reporting summary

Further information on research design is available in the Nature Portfolio Reporting Summary linked to this article.

## Online content

Any methods, additional references, Nature Portfolio reporting summaries, source data, extended data, supplementary information, acknowledgements, peer review information; details of author contributions and competing interests; and statements of data and code availability are available at 10.1038/s41593-024-01607-5.

## Supplementary information

Supplementary Information Supplementary Figs. 1–13 and Supplementary Notes 1–4

Reporting Summary

## Data Availability

All weights for language transformers used in this study were taken from pretrained models available on the Huggingface repository (https://huggingface.co/docs/transformers/). Training data for simulated psychophysical tasks were generated using code available at https://github.com/ReidarRiveland/Instruct-RNN/. The full set of trained model weights for all results is available upon request.

## Code availability

All code used to train models and analyze results can be found at https://github.com/ReidarRiveland/Instruct-RNN/.
