# Supplementary material for: Natural language instructions induce compositional generalization in networks of neurons
Source: Nat Neurosci. 2024 Mar 18;27(5):988–99. doi: 10.1038/s41593-024-01607-5 (PMC11537972; doi:10.1038/s41593-024-01607-5)
Supplement: Supplementary file 2 — Reporting Summary [file 41593_2024_1607_MOESM2_ESM.pdf]

nature portfolio

Corresponding author(s): Reidar Riveland

Last updated by author(s): Jan 18, 2024

Reporting Summary

Nature Portfolio wishes to improve the reproducibility of the work that we publish. This form provides structure for consistency and transparency in reporting. For further information on Nature Portfolio policies, see our Editorial Policies and the Editorial Policy Checklist.

Statistics

For all statistical analyses, confirm that the following items are present in the figure legend, table legend, main text, or Methods section.

n/a	Confirmed	
☐	☒	The exact sample size (*n*) for each experimental group/condition, given as a discrete number and unit of measurement
☐	☒	A statement on whether measurements were taken from distinct samples or whether the same sample was measured repeatedly
☐	☒	The statistical test(s) used AND whether they are one- or two-sided<br>*Only common tests should be described solely by name; describe more complex techniques in the Methods section.*
☒	☐	A description of all covariates tested
☒	☐	A description of any assumptions or corrections, such as tests of normality and adjustment for multiple comparisons
☐	☒	A full description of the statistical parameters including central tendency (e.g. means) or other basic estimates (e.g. regression coefficient) AND variation (e.g. standard deviation) or associated estimates of uncertainty (e.g. confidence intervals)
☐	☒	For null hypothesis testing, the test statistic (e.g. *F*, *t*, *r*) with confidence intervals, effect sizes, degrees of freedom and *P* value noted<br>*Give P values as exact values whenever suitable.*
☒	☐	For Bayesian analysis, information on the choice of priors and Markov chain Monte Carlo settings
☒	☐	For hierarchical and complex designs, identification of the appropriate level for tests and full reporting of outcomes
☐	☒	Estimates of effect sizes (e.g. Cohen's *d*, Pearson's *r*), indicating how they were calculated

Our web collection on statistics for biologists contains articles on many of the points above.

Software and code

Policy information about availability of computer code

Data collection	Data was generated in simulation using Python 3.7.11. PyTorch 1.10 was used to implement and train models (this includes Adam optimizer implementation). Transformers 4.16.2 was used to implement language models and all pre-trained weights for language models were taken from Huggingface repository (https://huggingface.co/docs/transformers/). Training, analysis, and plotting scripts available at https://github.com/ReidarRiveland/Instruct-RNN.
Data analysis	Data analysis performed in Python 3.7.11 (https://github.com/ReidarRiveland/Instruct-RNN). We also used scikit-learn 0.24.1 and scipy 1.7.3 to perform analyses.

For manuscripts utilizing custom algorithms or software that are central to the research but not yet described in published literature, software must be made available to editors and reviewers. We strongly encourage code deposition in a community repository (e.g. GitHub). See the Nature Portfolio guidelines for submitting code & software for further information.

Data

Policy information about availability of data

All manuscripts must include a data availability statement. This statement should provide the following information, where applicable:

- Accession codes, unique identifiers, or web links for publicly available datasets
- A description of any restrictions on data availability
- For clinical datasets or third party data, please ensure that the statement adheres to our policy

All weights for language transformers used in this study were taken from pre-trained models available on the Huggingface repository (https://huggingface.co/docs/transformers/). Training data for simulated psychophysical tasks was generated using code available at https://github.com/ReidarRiveland/Instruct-RNN. The full set of trained model weights for all results included in the manuscript is available upon request.

Research involving human participants, their data, or biological material

Policy information about studies with human participants or human data. See also policy information about sex, gender (identity/presentation), and sexual orientation and race, ethnicity and racism.

Reporting on sex and gender	N/A
Reporting on race, ethnicity, or other socially relevant groupings	N/A
Population characteristics	N/A
Recruitment	N/A
Ethics oversight	N/A

Note that full information on the approval of the study protocol must also be provided in the manuscript.

Field-specific reporting

Please select the one below that is the best fit for your research. If you are not sure, read the appropriate sections before making your selection.

☒ Life sciences ☐ Behavioural & social sciences ☐ Ecological, evolutionary & environmental sciences

For a reference copy of the document with all sections, see nature.com/documents/nr-reporting-summary-flat.pdf

Life sciences study design

All studies must disclose on these points even when the disclosure is negative.

Sample size	In cases applicable we analyzed the results from models that were trained across five different random initialization of parameters. This choice was constrained by computational feasibility but is on par with other computational neuroscience studies (e.g. Yang et. al., Nature Neuroscience, 2019).
Data exclusions	None
Replication	Models tested across the five different initializations yielded consistent results throughout experiments, both in performance and in analysis of model representations. Exact random seeds are provide in code such that training and testing can be reproduced exactly.
Randomization	Again, in the case of each model we used five different random initialization. The same set of five random seeds were used across each of these conditions.
Blinding	We did not use explicit blinding in the analysis and simulation was not performed blind to conditions. This is because all experiments were executed automatically in computer code. All model weights that were not part of pre-trained language models were randomly initialized, in which case the authors had no control over these initializations.

Reporting for specific materials, systems and methods

We require information from authors about some types of materials, experimental systems and methods used in many studies. Here, indicate whether each material, system or method listed is relevant to your study. If you are not sure if a list item applies to your research, read the appropriate section before selecting a response.

Materials & experimental systems

n/a	Involved in the study
☒	☐ Antibodies
☒	☐ Eukaryotic cell lines
☒	☐ Palaeontology and archaeology
☒	☐ Animals and other organisms
☒	☐ Clinical data
☒	☐ Dual use research of concern
☒	☐ Plants

Methods

n/a	Involved in the study
☒	☐ ChIP-seq
☒	☐ Flow cytometry
☒	☐ MRI-based neuroimaging

Plants

Seed stocks	N/A
Novel plant genotypes	N/A
Authentication	N/A
